# Insertion of CO_2_, Isocyanates, and Acetonitrile into the P–Si Bond of a Silyl-Substituted *N*‑Heterocyclic Carbene Phosphinidene

**DOI:** 10.1021/acs.organomet.5c00466

**Published:** 2026-02-13

**Authors:** Andreas Hochholzer, Martin E. Doleschal, Priyanka Chakraborty, Shigeyoshi Inoue

**Affiliations:** TUM School of Natural Sciences, Department of Chemistry, Catalysis Research Center and Institute of Silicon Chemistry, 163254 Technical University of Munich, Garching bei München 85748, Germany

## Abstract

This work investigates the reactivity of IDippP-SiMe_3_ (IDipp = 1,3-bis­(2,6-diisopropylphenyl)-imidazolin-2-ylidene) toward CO_2_, Ph-NCO, and Me-CN. Whereas carbon dioxide and phenyl isocyanate readily insert into the polarized P–Si bond, the activation of acetonitrile only proceeds via a frustrated Lewis pair (FLP) pathway in the presence of group 13 halides. Nitrile insertion in the presence of aluminum halides affords both an *N*-heterocyclic carbene phosphinidene (NHCP)-substituted imine and a phosphaalkene, with the product ratio depending on the choice of Lewis acid. In contrast, the addition of BCl_2_ *^m^* Ter (*^m^* Ter = 2,6-(2,4,6-methyl-C_6_H_2_)-C_6_H_3_) selectively affords an NHCP-substituted imine via dehalosilylation.

## Graphical abstract

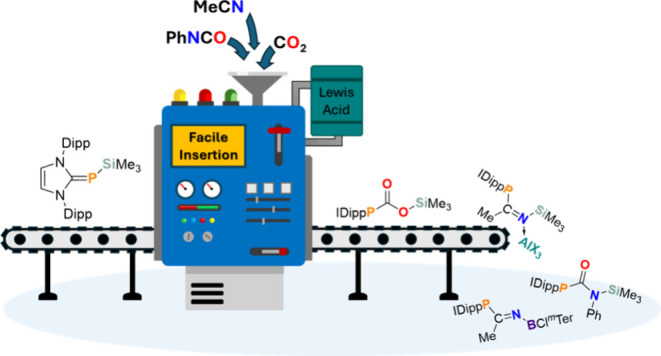

## Introduction

The activation of small molecules by main-group element compounds represents a central research area in modern catalysis and synthesis.^1,2^ In recent decades, various low-coordinate s- and p-block elements have been implemented to activate not only unsaturated hydrocarbons but also inert molecules such as H_2_ and NH_3_.^3–6^ Organophosphorus compounds have been established as versatile organocatalysts.^7^ While several phosphorus-based Lewis acid catalysts have been reported in the literature,^8^ due to the accessible electron lone pair of P­(III) species, they generally act as Lewis bases.^9^ In this context, phosphines have been employed in the activation of unsaturated hydrocarbons,^7^ as well as in the activation of inorganic substrates, including SO_2,_ NH_3,_ and CO_2._ ^10–12^ The nucleophilicity of phosphines renders them useful in frustrated Lewis pair (FLP) systems. After the seminal work of Stephan and co-workers, demonstrating the heterolytic splitting of dihydrogen,^13^ phosphine-based FLPs have been established for the activation of molecules such as SO_2_, NO_2_, CO, olefins, and nitriles, thereby further expanding their catalytic utility.^14–28^ *N*-Heterocyclic carbene-phosphinidenes (NHCPs) are an emerging class of organophosphorus compounds.^29,30^ Their major canonical forms describe them as inversely polarized phosphaalkenes (Scheme 1a, I & II).^31^ Yet, a potential dative C → P interaction^32,33^ (Scheme 1a, III) served as the basis for their name and renders them interesting compounds for studies on elusive phosphinidenes.^34^ Small molecule activation with NHCPs generally relies on the nucleophilic character of their exocyclic phosphorus atom; however, investigations in this field are limited to only a few examples, including the activation of O_2_, THF.^35–37^ Their utilization in organocatalysis was also investigated, demonstrating their ability to catalyze formylation and hydrodefluorination reactions, as well as the synthesis of γ-butyrolactones via nucleophilic and redox pathways.^38–40^ Formations of CO_2_ adducts have been reported with both phosphines and *N*-heterocyclic imines (NHIs), the nitrogen analogues of NHCPs (Scheme 1b).^41,42^ NHIs possess a more nucleophilic nitrogen with higher polarization, enhancing their electron-donating ability.^43,44^ In the presence of a SiMe_3_ substituent, insertions of CO_2_ and Isocyanates into the respective N–Si^45^ or P–Si^46^ bonds are observed. To the best of our knowledge, analogous reactivities have not yet been demonstrated with NHCPs. IDippP-SiMe_3_ ^47^ represents a promising candidate for these investigations, providing not only a Lewis basic phosphorus lone pair, but also the reactive P–Si bond. Furthermore, the bulky NHC scaffold promotes FLP-type reactivity by enhancing the frustration at the phosphorus center while preventing quenching by Lewis acids.^48^ We hypothesized that the highly polarized P–Si bond, combined with the steric protection present in our system, would allow for the activation of nitriles, which have been activated by intermolecular FLPs (Scheme 1c)^18–23^ but are generally inaccessible to conventional silylphosphine-based FLP systems.^49–51^ Herein, we report the activation of CO_2_ and Ph-NCO with IDippP-SiMe_3_, as well as a novel FLP-type insertion of acetonitrile.

**Scheme 1:**
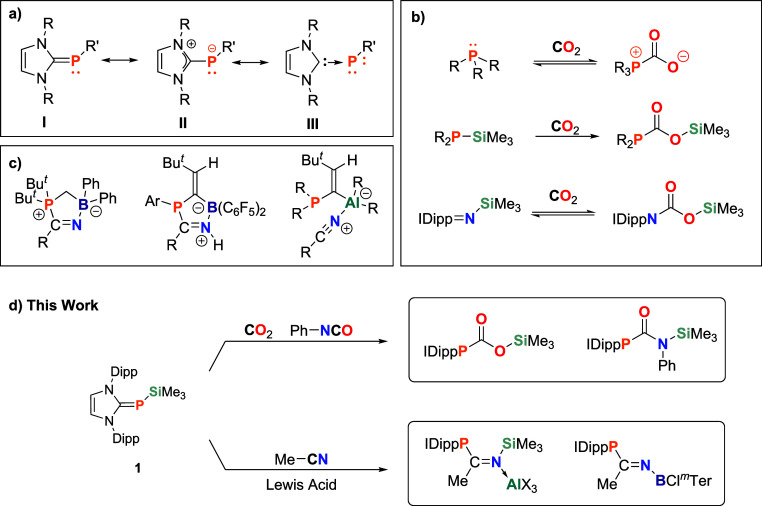
(a) Mesomeric Structures of NHCPs; (b) Selected Examples of CO_2_ Activation by Phosphines and NHIs; (c) Examples of Products Resulting from the Reaction of Phosphorus-Based FLPs with Nitriles; (d) Insertion of CO_2_, Ph-NCO, and Acetonitrile into the P–Si Bond of **1**

## Results and Discussion

Pressurising a solution of **1** with 1.0 bar of CO_2_ at room temperature results in the CO_2_ insertion into the silicon–phosphorus bond to obtain **2** (Scheme 2). It exhibits a ^31^P NMR resonance at −50.9 ppm, which is high-field shifted compared to insertion products derived from silylphosphines (−3.2 ppm),^52^ due to the increased electron density induced by the NHC. The ^29^Si NMR signal at 15.4 ppm is within the typical range of trimethylsiloxanes.^53^ We elucidated the structure of **2** via single crystal X-ray crystallography (SC-XRD), discovering C–O (1.398 (3) Å) and C=O (1.215 (3) Å) bond lengths, as well as the O–C–O bond angle (117.9 (2)°), which are in good agreement with phosphine-substituted esters (Figure 1).^54,55^ Compound **2** displays a C_NHC_–P bond length of 1.820 (2) Å, which is elongated compared to its precursor **1** (1.7744 (13) Å),^47^ and falls in the range of C–P single bonds.^30^ In contrast, the P–C_COO_ bond is considerably shorter (1.772 (3) Å), indicating the formation of a partial double bond (Scheme 2), which accounts for the irreversibility of the reaction. The reversible insertion of CO_2_ into a NHI-SiMe_3_ bond recently reported by Wilson et al.^56^ might be feasible due to an opposing trend in C_NHI_–N and N–C_COO_ bond lengths. Heating compound **2** leads to the formation of mainly IDippP-H among other decomposition products.

**Scheme 2:**
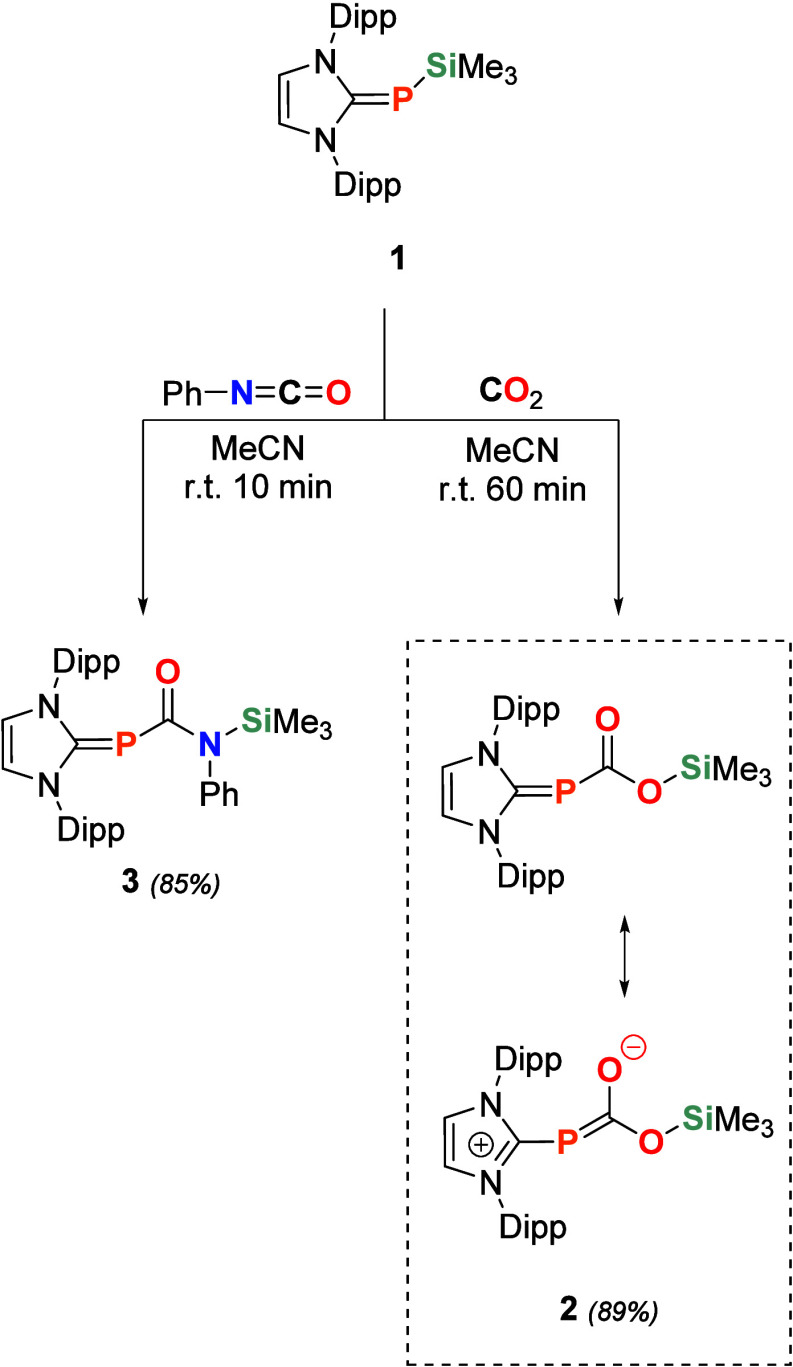
Reaction of **1** with CO_2_ and Ph-NCO, Forming **2** and **3**

**Figure 1:**
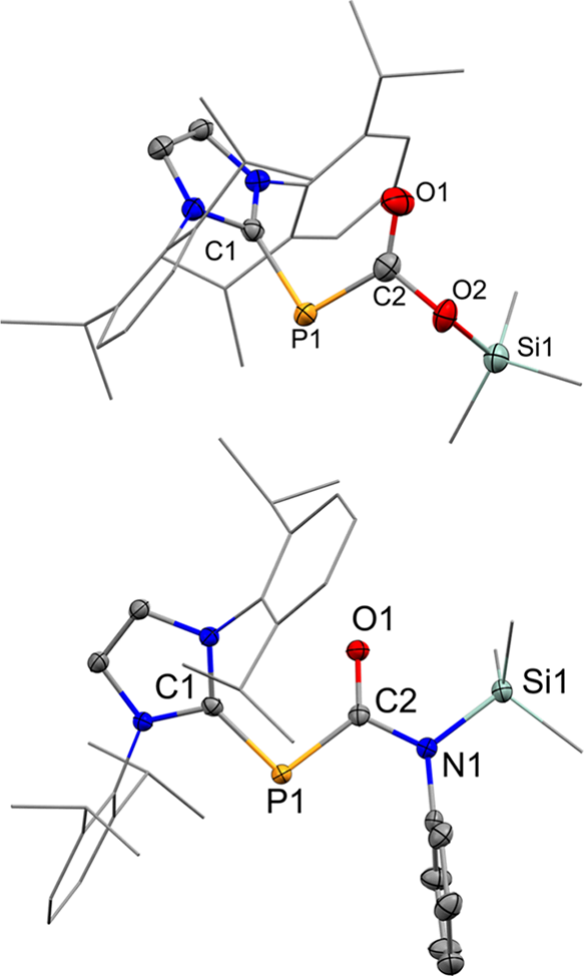
Solid-state plot of the molecular structure of **2** (top) and **3** (bottom). Thermal ellipsoids are set at the 50% probability. Hydrogen atoms have been omitted for clarity. Dipp and methyl substituents are depicted as wireframes for simplicity. Selected bond lengths (Å) and angles [°]: **2** (top): C1–P1 1.820 (2), P1–C2 1.772 (3), C1–O1 1.215 (3) C2–O2 1.398 (3), O2–Si1 1.664 (2), C1–P1–C2 94.5 (1), P1–C2–O1 129.9 (2), O1–C2–O2 117.9 (2), C2–O2–Si1 125.2 (2) **3** (bottom): C1–P1 1.789 (2), P1–C2 1.825 (1), C1–O1 1.237 (2), C2–N1 1.397 (2), N1–C3 1.434 (2), N1–Si1 1.775 (1), C1–P1–C2 98.43 (6), P1–C2–O1 127.2 (1), P1–C2–N1 115.7 (1), and C2–N1–Si1 115.5 (1).

Analogous to CO_2_, the insertion of isocyanates into P–Si and N–Si bonds is well studied.^57,58^ Exposure of **1** to 1.0 equiv of Ph-NCO results in the rapid formation of **3** (Scheme 2). The short reaction time reveals the significantly higher reactivity of IDippP-SiMe_3_ compared to Ph_2_P-SiMe_3_, for which the same reaction requires 3 days.^57^ Similar to **2**, the observed ^31^P NMR resonance of **3** (−30.7 ppm) is high-field shifted compared to its diphenylphosphine analogue.^59^ The ^29^Si NMR signal at 2.3 ppm is in line with reported silylamines.^60^ Single crystals of **3** (Figure 1) reveal a C_NHC_-P bond length of 1.789 (2) Å, which falls within the expected range of NHC=P double bonds,^30^ whereas the P–C_CON_ bond (1.825 (1) Å) is shorter than in related phosphorus-substituted amines.^61^ The C=O bond length of 1.237 (2) Å is characteristic of amides; however, the C–N bond length of 1.397 (2) Å is notably elongated.^62^ Insertion of isocyanates into P–Si bonds results in the formation of a new N–Si bond, which can be capable of reacting with a second isocyanate molecule.^63^ Neither in our case nor in previously reported silylphosphines^57^ was such a double insertion observed. Notably, these results differ from a recent finding that in the presence of a Ge­(II)–Si bond, phenyl isocyanate forms isocyanurate rather than inserting into the bond.^64^

To investigate whether IDippP-SiMe_3_ (**1**) can react in an FLP-type reaction, we tested its reactivity toward nitriles in the presence of different Lewis acids (Scheme 3). To compare **1** with conventional phosphines, we carried out control experiments and tried to activate acetonitrile with Ph_2_P-SiMe_3_ and P­(SiMe_3_)_3_. Regardless of the Lewis acid used, insertion reactions were not observed. However, after dropwise addition of **1** to a solution of stoichiometric amounts of acetonitrile and aluminum­(III)­chloride in diethyl ether, compound **4a** was obtained as a white precipitate. Its ^31^P NMR signal (88.2 ppm) is notably low-field shifted, compared to those of previously investigated insertion products (**2**: −50.9 ppm; **3**: −30.7 ppm). The ^29^Si NMR resonance of the SiMe_3_ group appears at 7.2 ppm (d, ^3^ *J*_Si–P_ = 12.7 Hz). Bright orange crystals of **4a** suitable for SC-XRD measurement were obtained from an acetonitrile solution stored at −35 °C (Figure 2). The C=N bond in **4a** measures 1.386 (2) Å and is elongated compared to similar imine adducts (1.284–1.335 Å).^65,66^ Consequently, the dative N → Al dative bond (1.859 (2) Å) falls below the range of bond lengths reported in the literature (1.874–2.003 Å).^67,68^ The elongated C_NHC_–P bond (1.837 (2) Å) and shortened P–C_CN_ bond (1.722 (2) Å) suggest a significant electron delocalization across the molecule (Scheme 3). Upon substitution of AlCl_3_ with AlBr_3_, the expected product **4b** is formed. **4b** exhibits a ^29^Si NMR signal at 6.8 ppm (d, ^3^ *J*_Si–P_ = 12.1 Hz), which is in good agreement with **4a**, while its ^31^P NMR resonance (102.4 ppm) was observed in slightly lower fields. Finally, using AlI_3_ as the Lewis acid led to the formation of an inseparable product mixture. The ^31^P NMR suggests at least partial formation of the **4a**/**4b** analogue; however, all other spectra revealed that it contains mostly decomposition products.

**Scheme 3:**
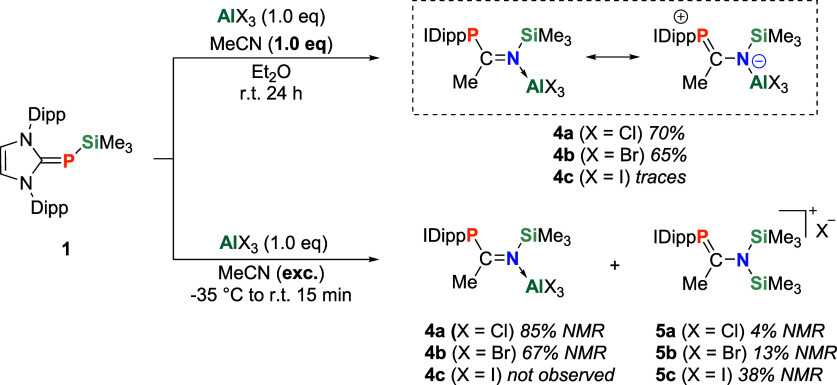
Reaction of **1** with Acetonitrile in the Presence of AlX_3_ (X = Cl, Br, I), Affording **4ab/5ab** and **5c**

**Figure 2:**
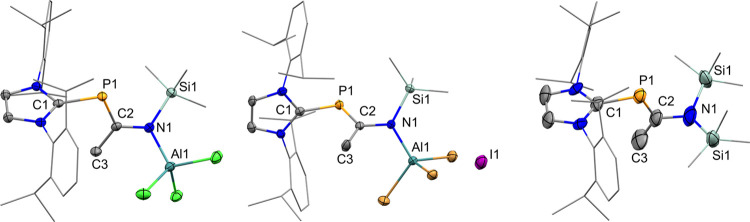
Solid-state plot of the molecular structure of **4ab/5c**. Thermal ellipsoids are set at 50% probability. Hydrogen atoms have been omitted for clarity. Dipp and methyl substituents are depicted as wireframes for simplicity. Selected bond lengths (Å) and angles (°): **4a** (left): C1–P1 1.837 (2), P1–C2 1.722 (2), C2–C3 1.500 (3), C2–N1 1.386 (2), N1–Si1 1.768 (2), N1–Al1 1.859 (2), C1–P1–C2 101.97 (8), P1–C2–C3 125.7 (1), C3–C2–N1 116.1 (2), P1–C2–N1 118.2 (1). **4b** (center): C1–P1 1.833 (4), P1–C2 1.727 (4), C2–C3 1.491 (6), C2–N1 1.382 (5), N1–Si1 1.788 (3), N1–Al1 1.862 (3) C1–P1–C2 102.9 (2), P1–C2–C3 124.9 (3), C3–C2–N1 116.9 (3), P1–C2–N1 118.2 (3). **5c** (right): C1–P1 1.833 (4), P1–C2 1.686 (4), C2–C3 1.501 (7), C2–N1 1.428 (5), N1–Si1 1.862 (2), C1–P1–C2 103.76 (19), P1–C2–C3 128.8 (3), P1–C2–N1 114.8 (3),C3–C2–N1 116.3 (4).

When acetonitrile is used as the solvent and, therefore, available in vast excess, monitoring the syntheses of **4a**/**4b** via ^31^P NMR reveals the formation of byproducts **5a**/**5b**, respectively. The ratio appears to be dependent on the Lewis acid and increases from 4% **5a** to 13% **5b**. Whereas the reaction of **1** with AlI_3_ in acetonitrile also failed to yield the desired nitrile insertion product, the byproduct (**5c**) was observed in about 38% yield. Notably, when **4a/4b** are redissolved in acetonitrile in the presence of **1**, trace amounts of **5a/5b** are formed. The low-field shifted ^31^P NMR signals of **5a**-**c** (137.0 (q, ^3^ *J*_Si–P_ = 8.7 Hz), 137.0 (q, ^3^ *J*_Si–P_ = 8.7 Hz), 137.2 (q, ^3^ *J*_Si–P_ = 10.0 Hz) ppm, respectively) fall within the range of similar NHC-substituted phosphaalkenes.^69^ Their ^29^Si NMR resonances (8.0 (d, ^3^ *J*_Si–P_ = 9.8 Hz), 8.0 (d, ^3^ *J*_Si–P_ = 10.3 Hz), and 8.0 ppm (d, ^3^ *J*_Si–P_ = 9.7 Hz), respectively) show negligible differences to **4** **a** **/4b**. Single crystals suitable for XRD analysis could be obtained only from **5c** (Figure 2). Its geometry reveals a P=C double bond (1.686 (4) Å) whose length lies at the lower end of the range for reported phosphaalkenes (1.67–1.71 Å).^70^ As expected for a sp^2^-hybridized carbon, the P=C bond adopts a planar structure (N–C–P–C: 180°). We propose that the Increasing ratio of byproducts **5a**–**5c** from chloride to iodide originates from the weakening of the dative N–Al bond, which in turn reflects the decreasing Lewis acidity of the corresponding aluminum halides.^71^ The formation of **5a**–**5c** requires quenching of an additional equivalent of **1**, whose decomposition product was identified as an imidazolium salt (see supporting info). However, due to the intrinsic reactivity of aluminum halides with acetonitrile,^72,73^ it is difficult to elucidate a precise reaction mechanism. Control experiments revealed that, in the absence of acetonitrile, the combination of **1** and AlCl_3_ leads to decomposition and thus prevents the characterization of a stable FLP. Notably, further steric shielding using AlCl_2_ *^m^* Ter inhibited the activation of the nitrile.

We anticipated that due to strong N–B bond formation and thus facile dehalosilylation, the usage of boron instead of aluminum halides could be beneficial. However, upon reacting them with **1**, only decomposition was observed. In contrast, adding a solution of **1** in acetonitrile to a stirred solution of *^m^* Ter substituted boron chloride led to the selective formation of imine **6** (Scheme 4). Unlike the aluminum-based system, the combination of **1** and BCl_2_ *^m^* Ter in the absence of acetonitrile does not lead to decomposition, confirming the formation of a frustrated Lewis pair. Analytically pure **6** was obtained after stirring the solution for 15 min at room temperature and subsequently cooling it to −35 °C overnight. Its ^31^P NMR shift (−0.36 ppm) can be observed within the range of typical phosphines.^200^ The high-field shift in comparison to **4** indicates significantly increased π–π interaction between carbon and the two-coordinate nitrogen, consistent with the shorter C–N (1.277 (4) Å) bond in **6** (Figure 3) compared to **4**. The ^11^B NMR signal at 27.55 ppm is in agreement with those of other iminoboranes.^74^ In contrast to the reported IDipp = NB­(Cl)­Ph (N–B = 1.350 (3) Å, C–N–B = 131.7 (2)°),^74^ compound **6** adopts a linear geometry with a C–N–B angle of 175.3 (3)° and a shorter N–B length (1.335 (4) Å), attributed to the stronger N–B π interaction.

**Figure 3:**
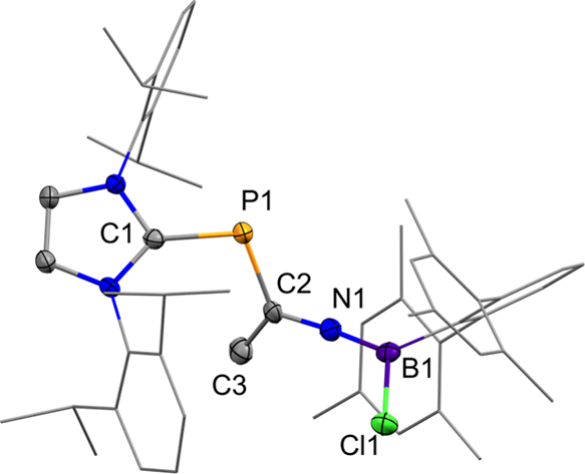
Solid-state plot of the molecular structure of **6**. Thermal ellipsoids are set at 50% probability. Hydrogen atoms have been omitted for clarity. Dipp and mesityl substituents are depicted as wireframes for simplicity. Selected bond lengths [Å] and angles [°]: C1–P1 1.788 (3), P1–C2 1.796 (3), C2–C3 1.506 (4), C2–N1 1.277 (4), N1–B1 1.335 (4), B1–Cl1 1.828 (3), B1–C4 1.587 (5), C1–P1–C2 105.2 (1), P2–C2–C3 123.5 (2), C3–C2–N1 118.7 (3), C2–N1–B1 175.3 (3).

**Scheme 4:**
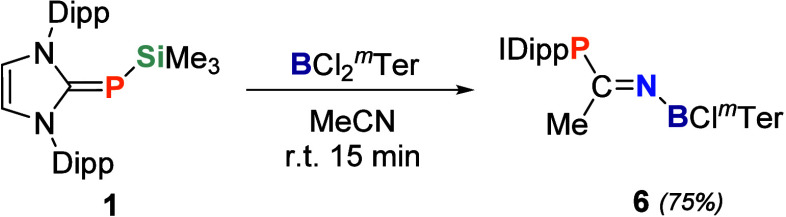
FLP-Type Insertion of Acetonitrile with BCl_2_ *^m^* Ter to Afford 6

## Conclusions

We demonstrated the reactivity of IDippP-SiMe_3_ (**1**) toward CO_2_, Ph-NCO, and Me-CN. Our studies revealed that **1** selectively inserts CO_2_ and Ph-NCO into its P–Si bond to afford compounds **2** and **3**, respectively. In the presence of Lewis acidic group 13 halides, compound **1** was able to insert acetonitrile as well, displaying higher reactivity compared to silylphosphines. In the presence of an excess of acetonitrile, depending on the halogen atoms, this reaction yielded NHCP-substituted imines (**4a/b**) and phosphaalkenes (**5a/b/c**) in different ratios. Finally, the reaction of **1** with BCl_2_ *^m^* Ter selectively affords the NHCP-substituted imine **6**, featuring a covalent N–B bond.

## Supplementary Material



## Notes

Experimental details, including full synthetic, spectroscopic data for **2**, **3**, **4a/b**, **5a/b/c**, IDippAlI_4_, and crystallographic data for **2**, **3**, **4a/b**, **5c**, and IDippAlI_4_ (PDF)
