# Supplementary material for: Insertion of CO2, Isocyanates, and Acetonitrile into the P–Si Bond of a Silyl-Substituted N‑Heterocyclic Carbene Phosphinidene
Source: Organometallics. 2026 Feb 13;45(5):573–9. doi: 10.1021/acs.organomet.5c00466 (PMC12977050; doi:10.1021/acs.organomet.5c00466)
Supplement: Supplementary file 1 [file om5c00466_si_001.pdf]

Supporting Information

Insertion of CO_2, Isocyanates, and Acetonitrile into the P–Si Bond of a Silyl-Substituted N-Heterocyclic Carbene-Phosphinidene

*Andreas Hochholzer, Martin E. Doleschal, Priyanka Chakraborty, Shigeyoshi Inoue**

s.inoue@tum.de

Content

Experimental Section ... S-3
General Procedures ... S-3
Chemicals & Materials ... S-3
NMR Spectroscopy ... S-3
Mass Spectroscopy ... S-3
Melting Point ... S-3
Elemental Analysis ... S-3
Single Crystal XRD Analysis ... S-4
Synthetic Procedures ... S-5
Synthesis of IDippP[CO_2]$SiMe_3$ (2) ... S-5
Synthesis of IDippP[PhNCO]$SiMe_3$ (3) ... S-6
Synthesis of IDippP[MeCN][$AlCl_3$]$SiMe_3$ (4a) ... S-7
Synthesis of IDippP[MeCN][$AlBr_3$]$SiMe_3$ (4b) ... S-8
Synthesis of IDippP[MeCN][$SiMe_3$]$_2$Cl (5a) ... S-9
Synthesis of IDippP[MeCN][$SiMe_3$]$_2$Br (5b) ... S-10
Synthesis of IDippP[MeCN][$SiMe_3$]$_2$I (5c) ... S-11
Synthesis of IDippP[MeCN][BClmTer] (6) ... S-12
IDippAlI_4 (by-product of 5c) ... S-13
Spectra ... S-14
Crystallographic Details ... S-37
References ... S-40

Experimental Section

General Procedures

Chemicals & Materials

All experiments were conducted under a dry argon (≥99.996%) atmosphere using standard Schlenk techniques or within a glovebox (MBraun GmbH). Glassware was dried under vacuum before use. Chemicals were purchased from ABCR GmbH, Carl Roth®, Merck KGaA, Sigma-Aldrich® and TCI Co. Ltd. and used without further purification. All solvents were stored over molecular sieve. Additionally, non-deuterated solvents were distilled over Calcium hydride. IDippP-$SiMe_3$ was synthesised according to literature procedures.[S1]

NMR Spectroscopy

NMR samples were prepared under argon atmosphere or in a glovebox and measured in J. Young PTFE valve NMR tubes. NMR spectra were recorded on Bruker AV-400 or AV-500C spectrometers at ambient temperature (300 K). ^{1}H, ^{13}C and ^{29}Si chemical shifts δ are reported in parts per million (ppm) relative to tetramethylsilane. δ(^{1}H) and δ(^{13}C) were referenced internally to the relevant residual solvent resonances. δ(^{29}Si) was referenced to the signal of tetramethylsilane (δ = 0 ppm) as external standard. The following abbreviations are used for reported signals: s = singlet, d = doublet, t = triplet, sept = septet, m = multiplet/signal overlap, br. = broad signal.

Mass Spectroscopy

Liquid Injection Field Desorption Ionisation Mass Spectrometry (LIFDI-MS) was measured directly from an inert atmosphere glovebox with a Thermo Fisher Scientific Exactive Plus Orbitrap equipped with an ion source from Linden CMS.[S2]

Melting Point

Melting points (m.p.) were determined in sealed glass capillaries under inert gas using a Büchi M-565 melting point apparatus.

Elemental Analysis

Elemental analyses were carried out by the microanalytical laboratory of the Catalysis Research Center, Technische Universität München.

Single Crystal XRD Analysis

The X-ray intensity data were collected on an X-ray single crystal diffractometer equipped with a CMOS detector (Bruker Photon-100), a rotating anode (Bruker TXS) with MoKα radiation (λ = 0.71073 Å) and a Helios mirror optic by using the APEX4 software package[S3] or an X-ray single crystal diffractometer equipped with a CMOS detector (Bruker Photon-100), an IMS microsource with MoKα radiation (λ = 0.71073 Å) and a Helios mirror optic by using the APEX4 software package.[S3] The measurement was performed on single crystals coated with perfluorinated ether. The crystal was fixed on the top of a microsampler, transferred to the diffractometer, and measured under a stream of cold nitrogen. A matrix scan was used to determine the initial lattice parameters. Reflections were merged and corrected for Lorenz and polarisation effects, scan speed, and background using SAINT.[S4] Absorption corrections, including odd and even ordered spherical harmonics were performed using SADABS.[S5] Space group assignments were based upon systematic absences, E statistics, and successful refinement of the structures. Structures were solved by direct methods with the aid of successive difference Fourier maps, and were refined against all data using the APEX4[S3] in conjunction with SHELXL-2017.[S6, 7] and SHELXLE.[S8] Methyl hydrogen atoms were refined as part of rigid rotating groups, with a C–H distance of 0.98 Å and Uiso(H) = 1.5·Ueq(C). Other H atoms were placed in calculated positions and refined using a riding model, with methylene and aromatic C-H distances of 0.99 and 0.95 Å, respectively, and Uiso(H) = 1.2·Ueq(C). If not mentioned otherwise, non-hydrogen atoms were refined with anisotropic displacement parameters. PLATON SQUEEZE was used to remove two highly disordered acetonitrile molecules and a diethylether molecule from crystal structures of 4b and 5c respectively.[S9] TWIN LAW (-1.0, 0.0, 0.0, 0.0, -1.0, 0.0, 0.0, 0.0, -1.0), BASF [0.027(11)] has been applied to refine a merohedral twinning in the crystal structure of 4b. Full-matrix least-squares refinements were carried out by minimising $\Delta w(Fo^2\text{-}Fc^2)^2$ with SHELXL-2014[S10] weighting scheme. Neutral atom scattering factors for all atoms and anomalous dispersion corrections for the non-hydrogen atoms were taken from International Tables for Crystallography.[S11] Images of the crystal structures were generated by MERCURY.[S12, 13] The CCDC numbers 2505556-2505562 contain the supplementary crystallographic data for the structures 2, 3, 4a, 4b, 5c, $IDippAlI_4$ and 6. These data can be obtained free of charge from the Cambridge Crystallographic Data Centre via https://www.ccdc.cam.ac.uk/structures/. The crystallographic information files (CIF) were generated using FinalCif.[S14]

Synthetic Procedures

Synthesis of IDippP[CO_2]$SiMe_3$ (2)

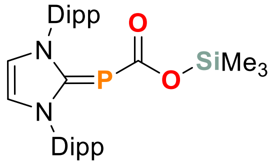

2

In a J. Young NMR tube IDippP-$SiMe_3$ (**1**, 20.0 mg, 40.59 mmol, 1.0 eq.) was dissolved in acetonitrile (0.4 mL) and exposed to 1.0 bar of CO_2. After one hour, all volatiles were removed in vacuo to afford **2** (crude) as a yellow solid. Crystals of **2** (89% 19.4 mg) suitable for XRD analysis could be grown from a diethyl ether solution stored at -35°C.

^{1}H NMR (400 MHz, CD_3CN): δ 7.48 (t, J = 7.8 Hz, 2H, *p*-Dipp), 7.41 (s, 2H, NC*H*), 7.33 (d, J = 7.9 Hz, 4H, *m*-Dipp), 2.67 (sept, J = 6.9 Hz, 4H, C*H*(CH_3)$_2$), 1.30 (d, J = 6.8 Hz, 12H, CH(CH_3)$_2$), 1.13 (d, J = 6.9 Hz, 12H, CH(CH_3)$_2$), -0.09 (s, 9H, Si(CH_3)$_3$).

^{13}C NMR (101 MHz, CD_3CN): δ 189.26 (d, $^{1}J_{P\text{-}C}$ = 47.6 Hz, O-*C*-O), 168.36 (d, $^{1}J_{C\text{-}P}$ = 91.2 Hz, ^{NHC}*C*-P), 146.93 (^{Dipp}Ar*C*), 135.02 (^{Dipp}*C*-N), 131.09 (^{Dipp}*p-C*), 125.05 (^{Dipp}*m-C*), 125.03 (N-*C*H), 29.68 (*C*H(CH_3)$_2$), 25.27 (CH(*C*H_3)$_2$), 23.24 (d, J = 2.5 Hz, CH(*C*H_3)$_2$), 0.42 (Si(*C*H_3)$_3$).

^{31}P NMR (162 MHz, CD_3CN): δ -50.90.

^{29}Si NMR (80 MHz, CD_3CN) δ 15.32 (d, $^{3}J_{Si\text{-}P}$ = 8.6 Hz).

LIFDI-MS: Calcd.: 536.2988
Found: 536.2967

m.p.: 115-120°C (no visible colour change)

Synthesis of IDippP[PhNCO]SiMe3 (3)

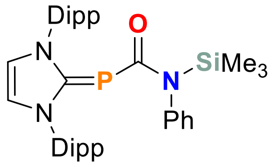

3

In a Schlenk flask **1** (20.0 mg, 40.59 mmol, 1.0 eq.) was dissolved in acetonitrile. Ph-NCO (4.41 µL, 40.59 µmol, 1.0 eq.) was added. Subsequently, the reaction mixture was stirred at room temperature for 10 minutes. All volatiles were removed in vacuo to afford **3** as a yellow solid. Crystals of **3** (85% 21.1 mg) suitable for XRD analysis could be grown from a hexane solution stored at -35°C.

1H NMR (400 MHz, CD3CN) δ 7.43 (t, *J* = 7.8 Hz, 2H, *p*-Dipp),), 7.30 (d, J = 7.9 Hz, 4H, *m*-Dipp), 7.26 (s, 2H, NC*H*), 7.10 (m, 1H,^{Ph}*p-H*), 7.08 (d, *J* = 2.3 Hz, 2H, ^{Ph}*o-H*), 6.81 – 6.72 (m, 2H, ^{Ph}*m-H*), 2.76 (sept, *J* = 6.9 Hz, 4H, C*H*(CH_3)$_2$), 1.30 (d, *J* = 6.9 Hz, 12H, CH(CH_3)$_2$), 1.11 (d, *J* = 7.0 Hz, 12H, CH(CH_3)$_2$), -0.24 (s, 9H, Si(CH_3)$_3$).

^{13}C NMR (101 MHz, CD_3CN) δ 194.67 (d, $^1J_{P\text{-}C}$ = 55.6 Hz, N*C*O), 171.50 (d, $^1J_{C\text{-}P}$ = 91.2 Hz, ^{NHC}*C*-P), 146.91 (^{Dipp}Ar*C*), 144.16 (d, $^3J_{C\text{-}P}$ = 6.2 Hz, ^{Ph}*C*-N), 135.63 (^{Dipp}*C*-N), 131.55 (d, *J* = 4.7 Hz, ^{Ph}*m-C*), 130.61(^{Dipp}*p-C*), 128.75 (^{Ph}*p-C*), 126.83 (^{Ph}*o-C*), 124.91 (^{Dipp}*m-C*), 124.21 (d, *J* = 2.5 Hz, N-*C*H), 29.69 (d, *J* = 1.5 Hz, *C*H(CH_3)$_2$), 25.24 (CH(*C*H$_3$)$_2$), 23.19 (d, *J* = 2.5 Hz, CH(*C*H$_3$)$_2$), 0.86 (Si(*C*H$_3$)$_3$).

^{31}P NMR (162 MHz, CD_3CN) δ -30.74.

^{29}Si NMR (80 MHz, CD_3CN) δ 2.28.

LIFDI-MS: Calcd.: 611.3461
Found: 611.3448

m.p.: 84-85°C (colour change to brown at around 100°C)

Synthesis of IDippP[MeCN][$AlCl_3$]$SiMe_3$ (4a)

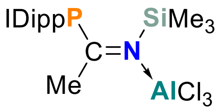

4a

In a Schlenk flask, acetonitrile (2.14 µL, 40.59 mmol, 1.0 eq.) and $AlCl_3$ (5.41 mg, 40.59 mmol, 1.0 eq.) were dissolved in diethyl ether and cooled to -35°C. Under vigorous stirring, a solution of IDippP-$SiMe_3$ (**1**, 20.0 mg, 40.59 mmol, 1.0 eq.) in diethyl ether was added dropwise. While the mixture was slowly warmed to room temperature, precipitation of **4a** was observed. After stirring for an additional 24h, the mixture was centrifuged, and the solvent was removed with a syringe. By removing the remaining solvent in vacuo, **4a** (19.0 mg, 70%) could be obtained as yellow/white solid. Crystals of **4a** suitable for XRD analysis could be grown from an acetonitrile solution stored at -35°C.

^{1}H NMR (400 MHz, CD_3CN) δ 7.87 (s, 2H, NC*H*), 7.59 (t, *J* = 7.8 Hz, 2H, *p*-Dipp), 7.44 (d, *J* = 7.9 Hz, 4H, *m*-Dipp), 2.48 (sept, *J* = 6.8 Hz, 4H, C*H*(CH_3)$_2$), 2.37 (d, $^{3}J_{H\text{-}P}$ = 7.0 Hz, 3H, CH_3-CN), 1.35 (d, *J* = 6.8 Hz, 12H, CH(CH_3)$_2$), 1.17 (d, *J* = 6.9 Hz, 12H CH(CH_3)$_2$), -0.06 (s, 9H, Si(CH_3)$_3$).

^{13}C NMR (126 MHz, CD_3CN) δ 225.83 (d, $^{1}J_{C\text{-}P}$ = 56.7 Hz, CH_3-*C*N), 156.89 (d, $^{1}J_{C\text{-}P}$ = 99.9 Hz, ^{NHC}*C*-P), 146.21 (^{Dipp}Ar*C*), 132.66 (^{Dipp}*C*-N), 132.61 (^{Dipp}*p-C*), 128.07 (d, *J* = 2.5 Hz, N-*C*H), 125.95 (^{Dipp}*m-C)*, 36.24 (d, $^{2}J_{C\text{-}P}$ = 6.3 Hz, *C*H_3-CN), 30.09 (d, *J* = 2.0 Hz, *C*H(CH_3)$_2$), 26.08 (CH(*C*H_3)$_2$), 22.59 (d, *J* = 3.0 Hz, CH(*C*H_3)$_2$), 2.37 (d, *J* = 6.5 Hz, Si(*C*H_3)).

Note: The *C*H_3-CN signal appears as a weak singlet. However, it was also detected via HSQC (see spectra) as a multiplet at 36.24 ppm.

^{31}P NMR (162 MHz, CD_3CN) δ 88.57.

^{29}Si NMR (99 MHz, CD_3CN) δ 7.17 (d, $^{3}J_{Si\text{-}P}$ = 12.3 Hz).

LIFDI-MS: Calcd.: 462.3038 [M +2H, - $AlCl_3$ - $SiMe_3$]$^+$
Found: 462.2995

m.p.: 150°C (slow colour change to orange)

Synthesis of IDippP[MeCN][AlBr$_3$]SiMe$_3$ (4b)

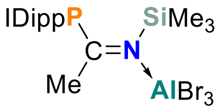

4b

In a Schlenk flask, acetonitrile (2.14 µL, 40.59 mmol, 1.0 eq.) and $AlCl_3$ (10.82 mg, 40.59 mmol, 1.0 eq.) were dissolved in diethyl ether and cooled to -35°C. Under vigorous stirring, a solution of IDippP-SiMe$_3$ (**1**, 20.0 mg, 40.59 mmol, 1.0 eq.) in diethyl ether was added dropwise. While the mixture was slowly warmed to room temperature, precipitation of **4b** was observed. After stirring for an additional 24h, the mixture was centrifuged, and the solvent was removed with a syringe. By removing the remaining solvent in vacuo, **4b** (21.1 mg, 65%) could be obtained as yellow/white solid. Crystals of **4b** suitable for XRD analysis could be grown from an acetonitrile solution stored at -35°C.

^{1}H NMR (400 MHz, CD_3CN) δ 7.88 (s, 2H, NC*H*), 7.58 (t, J = 7.8 Hz, 2H, *p*-Dipp), 7.44 (d, J = 7.9 Hz, 4H, *m*-Dipp), 2.47 (sept, J = 6.8 Hz, 4H, C*H*(CH$_3$)$_2$), 2.35 (d, $^3J_{H\text{-}P}$ = 7.8 Hz, 3H, CH_3-CN), 1.36 (d, J = 6.8 Hz, 12H, CH(CH_3)$_2$), 1.17 (d, J = 6.8 Hz, 12H, CH(CH_3)$_2$), -0.05 (s, 9H, Si(CH_3)$_3$).

^{13}C NMR (101 MHz, CD_3CN) δ 226.68 (d, $^1J_{C\text{-}P}$ = 53.0 Hz, CH$_3$-*C*N) 156.14 (d, $^1J_{C\text{-}P}$ = 101.4 Hz, ^{NHC}*C*-P), 146.18 (^{Dipp}Ar*C*), 132.71 (^{Dipp}*p-C*), 132.58 (^{Dipp}*C*-N), 128.22 (d, J = 2.9 Hz, N-*C*H), 126.06 (^{Dipp}*m-C)*, 37.47 (d, $^2J_{C\text{-}P}$ = 6.2 Hz, *C*H$_3$-CN), 30.17 (d, J = 2.5 Hz, *C*H(CH$_3$)$_2$), 26.18 (CH(*C*H$_3$)$_2$), 22.62 (d, J = 2.9 Hz, CH(*C*H$_3$)$_2$), 2.77 (d, J = 5.1 Hz, Si(*C*H$_3$)$_3$).

^{31}P NMR (162 MHz, CD_3CN) δ 102.67.

^{29}Si NMR (80 MHz, CD_3CN) δ 6.77 (d, $^3J_{Si\text{-}P}$ = 12.4 Hz).

LIFDI-MS: Calcd.: 462.3037 [M +2H, - AlBr$_3$ - SiMe$_3$]$^+$
Found: 462.2995

m.p.: 161°C (slow colour change to orange)

Synthesis of IDippP[MeCN][SiMe$_3$]$_2$Cl (5a)

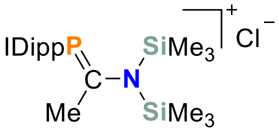

5a

In a Schlenk flask, $AlCl_3$ (5.41 mg 40.59 mmol, 1.0 eq.) was dissolved in acetonitrile and the solution was cooled to -35°C. Under vigorous stirring, a mixture of IDippP-SiMe$_3$ (**1**, 20.0 mg, 40.59 mmol, 1.0 eq.) in acetonitrile was added dropwise. After stirring for 15 min, all volatiles were removed in vacuo. The isolation of pure **5a** remained unsuccessful. The amount of **4a** could be lowered by extracting the product mixture with cold acetonitrile.

The reaction was carried out in 0.4 mL deuterated acetonitrile with mesitylene (18 mM) as an internal standard, to determine a yield of 4% via ^{1}H NMR**.**

^{1}**H NMR** (500 MHz, CD3CN) δ 8.00 (s, 2H, NC*H*), 7.62 (t, *J* = 7.8 Hz, 2H, *p*-Dipp), 7.47 (d, J = 7.8 Hz, 4H, *m*-Dipp), 2.52 – 2.43 (m, 4H, C*H*(CH$_3$)$_2$), 2.13 (d, $^{3}J_{H\text{-}P}$ = 9.6 Hz, 3H, C*H*$_3$-CN), 1.34 (d, J = 6.7 Hz, 12H, CH(C*H*$_3$)$_2$), 1.19 (d, J = 6.9 Hz, 12H, CH(C*H*$_3$)$_2$), -0.14 (s, 18H, Si(C*H*$_3$)$_3$).

Note: The aromatic and the ^{i}Pr-CH signals of the product overlap with **4a** and the imidazolium salt.

^{13}**C NMR** (126 MHz, CD$_3$CN) δ 223.74 (d, $^{1}J_{C\text{-}P}$ = 43.2 Hz, CH$_3$-*C*N), 153.03 (d, $^{1}J_{C\text{-}P}$ = 96.9 Hz, ^{NHC}*C*-P), 146.11 (^{Dipp}Ar*C*), 133.10 (^{Dipp}*p-C*), 131.78 (^{Dipp}*C*-N), 128.79 (d, *J* = 2.5 Hz, N-*C*H), 126.20 (^{Dipp}*m-C)* , 36.07 (d, $^{2}J_{C\text{-}P}$ = 8.0 Hz *C*H$_3$-CN), 30.20 (d, *J* = 2.5 Hz, *C*H(CH$_3$)$_2$), 26.22 (CH(*C*H$_3$)$_2$), 22.46 (d, *J* = 2.5 Hz, CH(*C*H$_3$)$_2$).

^{31}**P NMR** (203 MHz, CD$_3$CN) δ 137.01 (q, $^{3}J_{P\text{-}Si}$ = 8.7 Hz).

^{29}**Si NMR** (99 MHz, CD$_3$CN) δ 8.02 (d, $^{3}J_{Si\text{-}P}$ = 9.7 Hz).

LIFDI-MS: Calcd.: 462.3038 [M +2H, - 2SiMe$_3$]$^+$
Found: 462.3014

Note: The sample contained a mixture of products **4a** and **5a**. The spectra suggest that both compounds fragment to the same species.

Synthesis of IDippP[MeCN][SiMe3]2Br (5b)

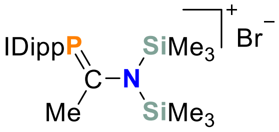

5b

In a Schlenk flask, $AlBr_3$ (10.82 mg 40.59 mmol, 1.0 eq.) was dissolved in acetonitrile and the solution was cooled to -35°C. Under vigorous stirring, a mixture of IDippP-$SiMe_3$ (**1**, 20.0 mg, 40.59 mmol, 1.0 eq.) in acetonitrile was added dropwise. After stirring for 15 min, all volatiles were removed in vacuo. The isolation of **5b** remained unsuccessful. The amount of **4b** could be lowered by extracting the product mixture with cold acetonitrile.

The reaction was carried out in 0.4 mL deuterated acetonitrile with mesitylene (18 mM) as an internal standard, to determine a yield of 13% via ^{1}H NMR**.**

^{1}H NMR (500 MHz, CD_3CN) δ 8.00 (s, 2H, NC*H*), 7.62 (t, *J* = 7.8 Hz, 2H, *p*-Dipp), 7.48 (d, *J* = 7.8 Hz, 4H, *m*-Dipp), 2.51 – 2.44 (m, 4H, C*H*(CH_3)$_2$), 2.13 (d, $^{3}J_{H\text{-}P}$ = 9.6 Hz, 3H, C*H*$_3$-CN), 1.35 (d, *J* = 4.8 Hz, 12H, CH(C*H*$_3$)$_2$), 1.20 (d, *J* = 6.7 Hz, 12H, CH(C*H*$_3$)$_2$), -0.14 (s, 18H, Si(C*H*$_3$)$_3$).

Note: The aromatic and the ^{i}Pr-CH signals of the product overlap with **4b** and the imidazolium salt.

^{13}C NMR (126 MHz, CD_3CN) δ 223.74 (d, $^{1}J_{C\text{-}P}$ = 43.2 Hz CH_3-*C*N), 153.04 (d, $^{1}J_{C\text{-}P}$ = 96.9 Hz, ^{NHC}*C*-P), 146.11 (^{Dipp}Ar*C*), 133.11 (^{Dipp}*p-C*), 131.78 (^{Dipp}*C*-N), 128.80 (d, *J* = 2.5 Hz, N-*C*H), 126.21 (^{Dipp}*m-C)*, 36.09 (d, $^{2}J_{C\text{-}P}$ = 7.5 Hz, *C*H$_3$-CN), 30.21 (d, *J* = 2.5 Hz, *C*H(CH_3)$_2$), 26.25 (CH(*C*H$_3$)$_2$), 22.49 (d, *J* = 2.5 Hz, CH(*C*H$_3$)$_2$), 1.75 (Si(*C*H$_{3)}$)).

^{31}P NMR (203 MHz, CD3CN) δ 136.99 (q, $^{3}J_{P\text{-}Si}$ = 8.7 Hz).

^{29}Si NMR (99 MHz, CD_3CN) δ 8.01 (d, $^{3}J_{Si\text{-}P}$ = 9.7 Hz).

LIFDI-MS: Calcd.: 462.3038 [M +2H, - 2SiMe$_3$]$^+$
Found: 462.3037

Note: The sample contained a mixture of products **4b** and **5b**. The spectra suggest that both compounds fragment to the same species.

Synthesis of IDippP[MeCN][SiMe$_3$]$_2$I (5c)

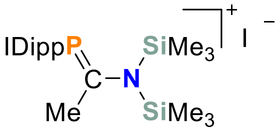

5c

In a Schlenk flask, AlI_3 (16.55 mg 40.59 mmol, 1.0 eq.) was dissolved in acetonitrile and the solution was cooled to -35°C. Under vigorous stirring, a mixture of IDippP-SiMe$_3$ (**1**, 20.0 mg, 40.59 mmol, 1.0 eq.) in acetonitrile was added dropwise. After stirring for 15 min, all volatiles were removed in vacuo. The separation **5c** from the imidazolium salt side product remained unsuccessful. Crystals of **5c** suitable for XRD analysis could be grown from a diethyl ether solution stored at -35°C.

The reaction was carried out in 0.4 mL deuterated acetonitrile with mesitylene (18 mM) as an internal standard, to determine a yield of 38% via ^{1}H NMR**.**

^{1}H NMR (500 MHz, CD_3CN) δ 8.10 (2H, NC*H*), 7.61 (t, *J* = 7.8 Hz, 2H, *p*-Dipp), 7.46 (d, *J* = 7.7 Hz, 4H, *m*-Dipp), 2.48 – 2.42 (m, 4H, C*H*(CH$_3$)$_2$), 2.11 (d, *J* = 9.6 Hz, 3H, C*H*$_3$-CN), 1.33 (d, *J* = 6.9 Hz, 12H, CH(C*H*$_3$)$_2$), 1.19 (d, *J* = 6.7 Hz, 12H, CH(C*H*$_3$)$_2$), -0.16 (s, 18H, Si(C*H*$_3$)$_3$).

Note: The aromatic and the ^{i}Pr-CH signals of the product overlap with the imidazolium salt.

^{13}C NMR (126 MHz, CD_3CN) δ 223.69 (d, $^{1}J_{C\text{-}P}$ = 43.2 Hz, CH$_3$-*C*N), 152.96 (d, $^{1}J_{C\text{-}P}$ = 96.9 Hz, ^{NHC}*C*-P), 146.12 (^{Dipp}Ar*C*), 128.97 (d, *J* = 2.5 Hz, N-*C*H), 126.92 (^{Dipp}*m-C)*, 36.09 (d, $^{2}J_{C\text{-}P}$ = 7.5 Hz, *C*H$_3$-CN), 30.21 (d, *J* = 2.5 Hz, *C*H(CH$_3$)$_2$), 26.31 (CH(*C*H$_3$)$_2$), 22.49 (d, *J* = 3.0 Hz, CH(*C*H$_3$)$_2$), 1.92 (Si(*C*H$_3$)).

^{31}P NMR (203 MHz, CD_3CN) δ 137.18 (q, $^{3}J_{P\text{-}Si}$ = 10.0 Hz).

^{29}Si NMR (99 MHz, CD_3CN) δ 7.94 (d, $^{3}J_{Si\text{-}P}$ = 10.3 Hz).

LIFDI-MS: Calcd.: 461.2960 [M +H, - 2SiMe$_3$]$^{+}$
Found: 461.3328

Synthesis of IDippP[MeCN][BClmTer] (6)

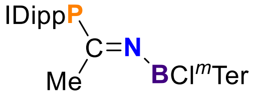

6

In a Schlenk flask, $BCl_2{}^{m}$Ter (16.0 mg 40.59 mmol, 1.0 eq.) was dissolved in acetonitrile. Under vigorous stirring, a mixture of IDippP-$SiMe_3$ (**1**, 20.0 mg, 40.59 mmol, 1.0 eq.) in acetonitrile was added dropwise. After stirring for 15 min, all volatiles were removed in vacuo. The remaining solid was washed with pentane and dried to afford **6** as an orange solid in 75% yield (23.30 mg). Crystals of **6** suitable for XRD analysis could be grown from a diethyl ether solution stored at -35°C.

^{1}H NMR (400 MHz, C_6D_6) δ 7.23 (t, J = 7.5 Hz, 1H, ^{mTer}*p*-C*H*), 7.09 (t, J = 7.7 Hz, 2H, ^{Dipp}*p*-C*H*), 6.99 (t, J = 7.7 Hz, 6H, ^{mTer}*m*-C*H*/^{Dipp}*m*-C*H*), 6.86 (s, 4H, ^{mTer}*m*-C*H*), 6.29 (s, 2H, NC*H*), 2.81 (sept, J = 6.9 Hz, 4H, C*H*(CH_3)$_2$), 2.26 (s, 6H, ^{mTer}*p*-C*H$_3$*), 2.24 (s, 12H, ^{mTer}*o*-C*H$_3$*), 1.31 (d, J = 6.8 Hz, 12H, CH(C*H*$_3$)$_2$), 1.24 (s, 3H, *H*$_3$C-CN), 0.98 (d, J = 6.9 Hz, 12H, CH(C*H*$_3$)$_2$).

^{13}C NMR (101 MHz, C_6D_6) δ 176.71 (d, $^{1}J_{C\text{-}P}$ = 57.4 Hz, CH_3-*C*N), 171.47 (d, $^{1}J_{C\text{-}P}$ = 110.3 Hz, ^{NHC}*C*-P), 145.68 (^{Dipp}Ar*C*), 144.83 (^{m}Ter), 141.44 (^{m}Ter), 136.75 (^{m}Ter), 135.26 (^{m}Ter), 133.56 (^{m}Ter), 130.98 (^{Dipp}*p-C*), 128.21 (^{m}Ter), 127.40 (^{m}Ter), 125.16 (^{Dipp}*m-C)*, 122.12 (d, J = 3.3 Hz, N-*C*H), 32.62 (d, $^{2}J_{C\text{-}P}$ = 2.4 Hz, *C*H$_3$-CN), 29.26 (d, J = 1.9 Hz, *C*H(CH_3)$_2$), 25.31 (CH(*C*H$_3$)$_2$), 22.74 (d, J = 2.4 Hz, CH(*C*H$_3$)$_2$), 21.64 (d, J = 2.4 Hz, ^{m}Ter), 21.32 (^{m}Ter).

^{31}P NMR (162 MHz, C_6D_6) δ -0.36.

^{11}B NMR (128 MHz, C_6D_6) δ 27.55.

LIFDI-MS: Calcd.: 419.2616 [M -BCl^{m}Ter]$^{+}$
Found: 419.261

Elemental analysis (%): Calcd: C 77.60; H 7.86; N 5.12; Found: C 74.95; H 7.85; N 5.00.

Note: The low value for carbon is explained by formation of incombustible boron carbides.

m.p.: 200°C (no visible colour change)

IDippAlI$_4$ (by-product of 5c)

The imidazolium salt side product formed during the synthesis of **5c** could be selectively synthesised via the reaction of NHC with AlI_3 in acetonitrile.

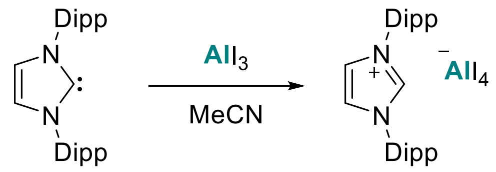

IDipp (20.0 mg, 51.20 mmol, 1.0 eq.) and AlI_3 (20.87 mg, 51.20 mmol, 1.0 eq.) were added to a Schlenk flask. The mixture was dissolved in acetonitrile and stirred for 24 hours at room temperature. Subsequently, all volatiles were removed in vacuo to obtain **IDippAlI$_4$** as an off-white solid (yield was not determined). Crystals of **IDippAlI$_4$** suitable for XRD analysis could be grown from a diethyl ether solution stored at -35°C.

^{1}**H NMR** (400 MHz, CD_3CN) δ 9.60 (t, *J* = 1.6 Hz, 1H), 7.94 (d, *J* = 1.6 Hz, 2H), 7.64 (t, *J* = 7.8 Hz, 4H), 7.46 (d, *J* = 7.9 Hz, 4H), 2.41 (p, *J* = 6.8 Hz, 4H), 1.26 (d, *J* = 6.8 Hz, 12H), 1.20 (d, *J* = 6.9 Hz, 12H).

^{13}**C NMR** (101 MHz, CD_3CN) δ 146.23, 133.03, 130.70, 126.91, 125.61, 29.82, 24.57, 23.78.

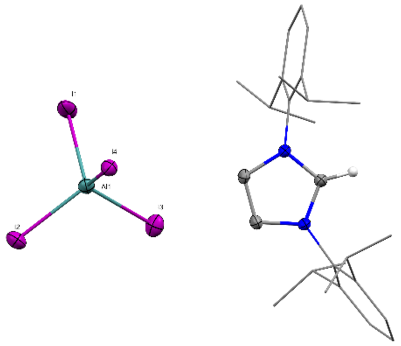

Figure S 1: Solid-state plot of the molecular structure of **IDippAlI$_4$** obtained from a diethyl ether solution stored at 35°C. Thermal ellipsoids are set at 50% probability. Hydrogen atoms are omitted for clarity.

Spectra

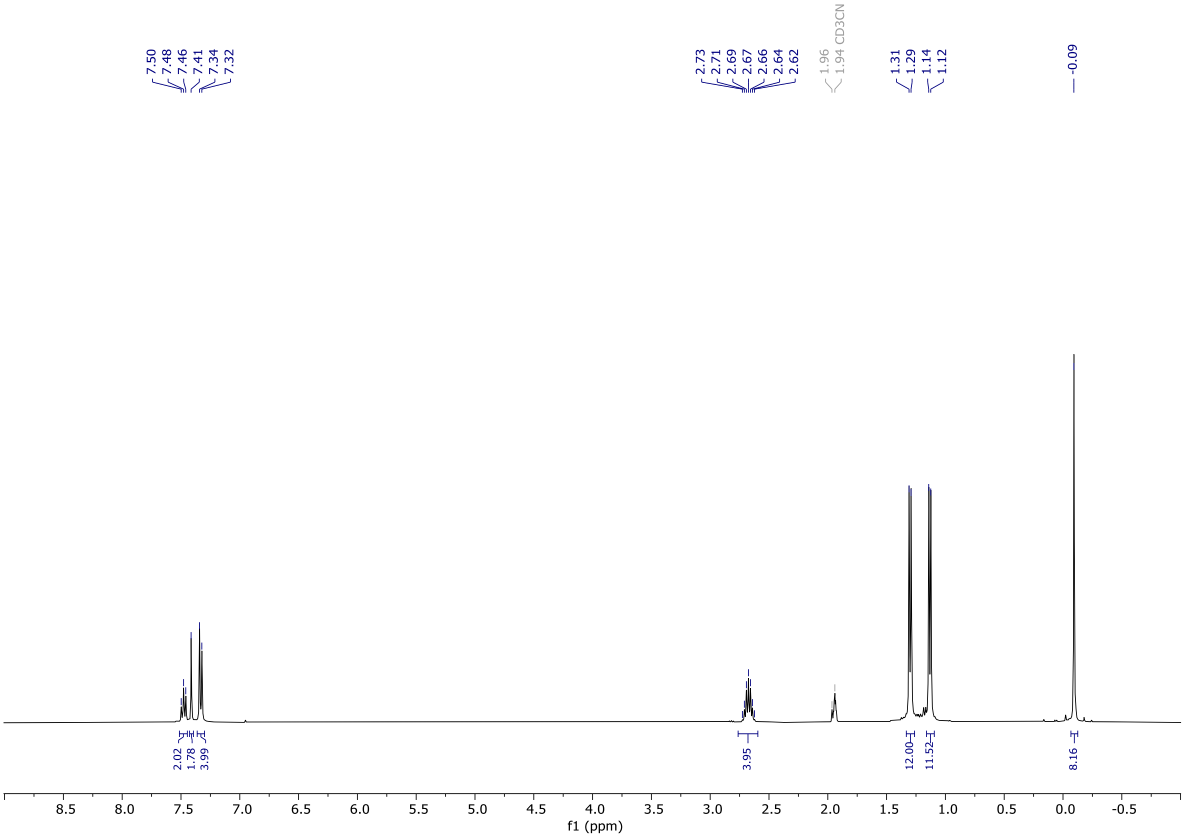

Figure S 2: ^{1}H NMR of compound **2**. The marked impurity (grey) is CH_3CN (1.94 ppm).

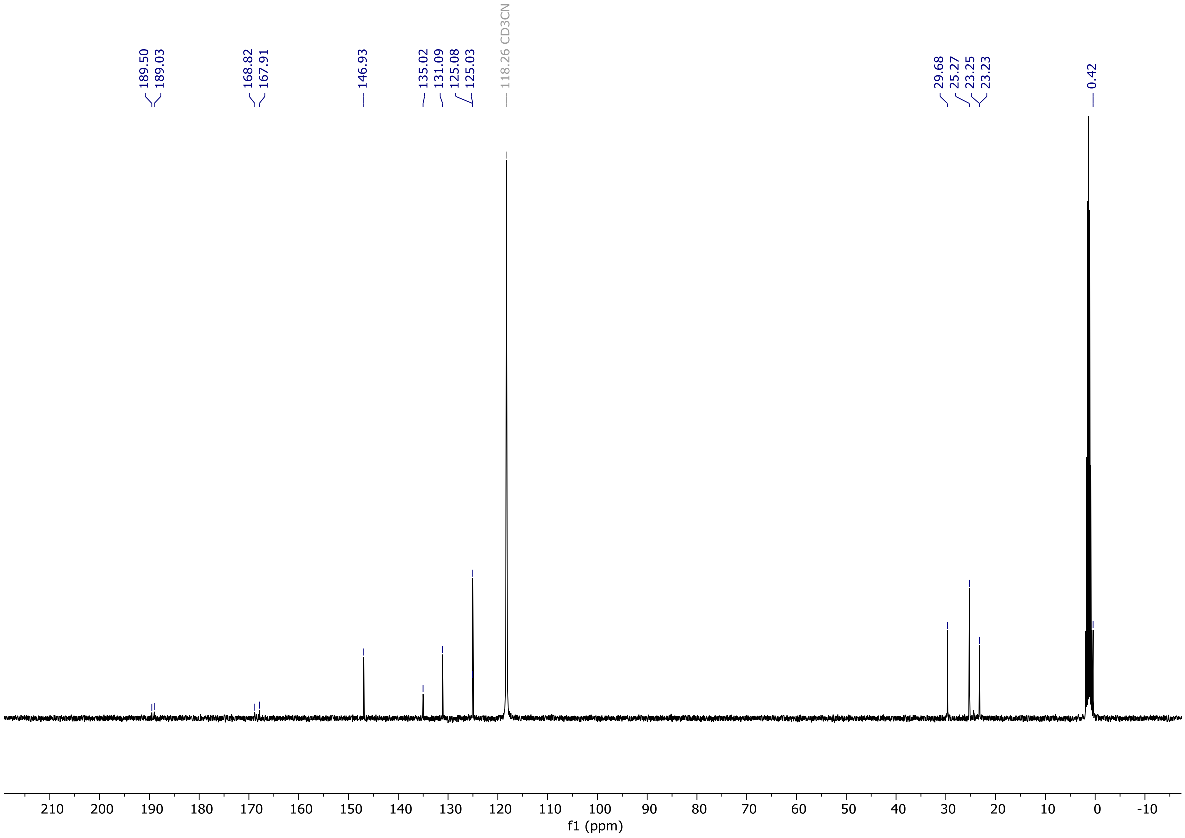

Figure S 3^{13}C NMR of compound **2**.

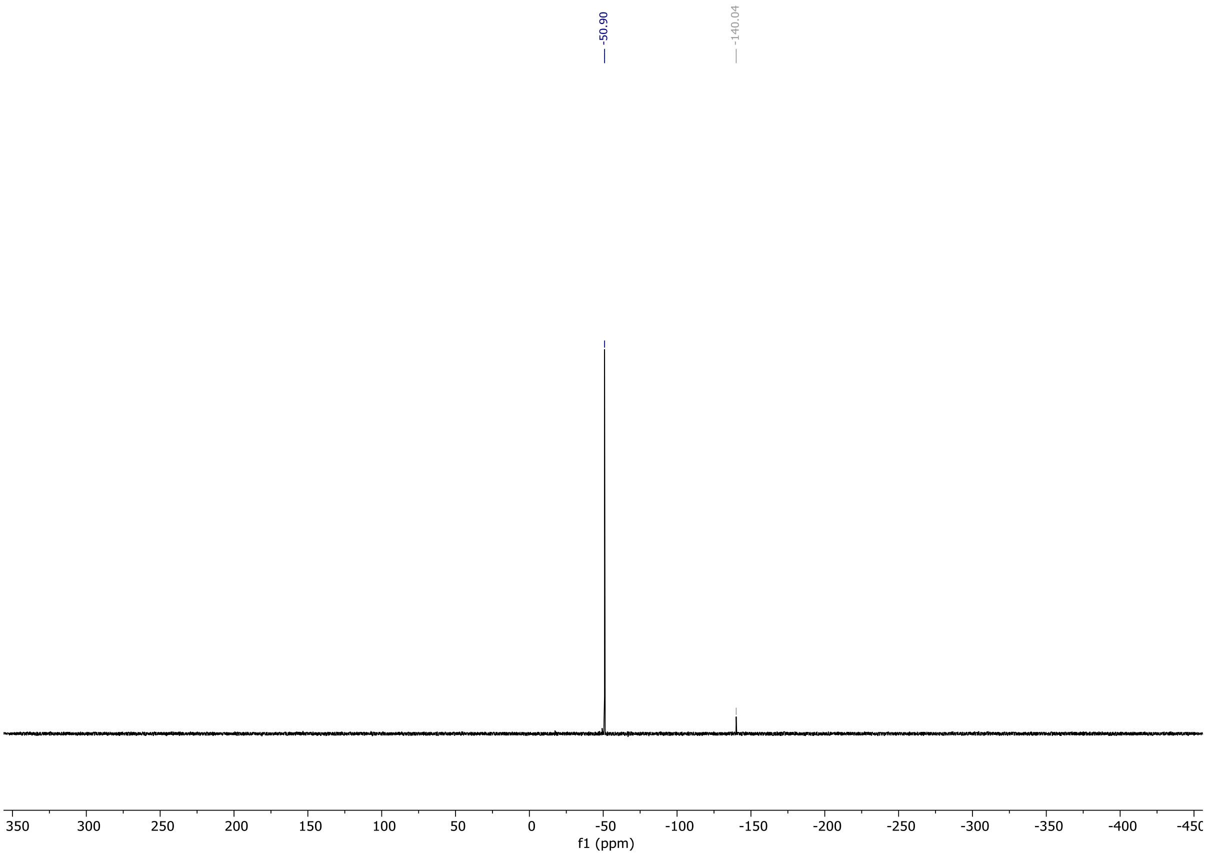

Figure S 4: ^{31}P NMR of compound **2**. The marked impurity (grey) is IDippP-H (-140.04 ppm).

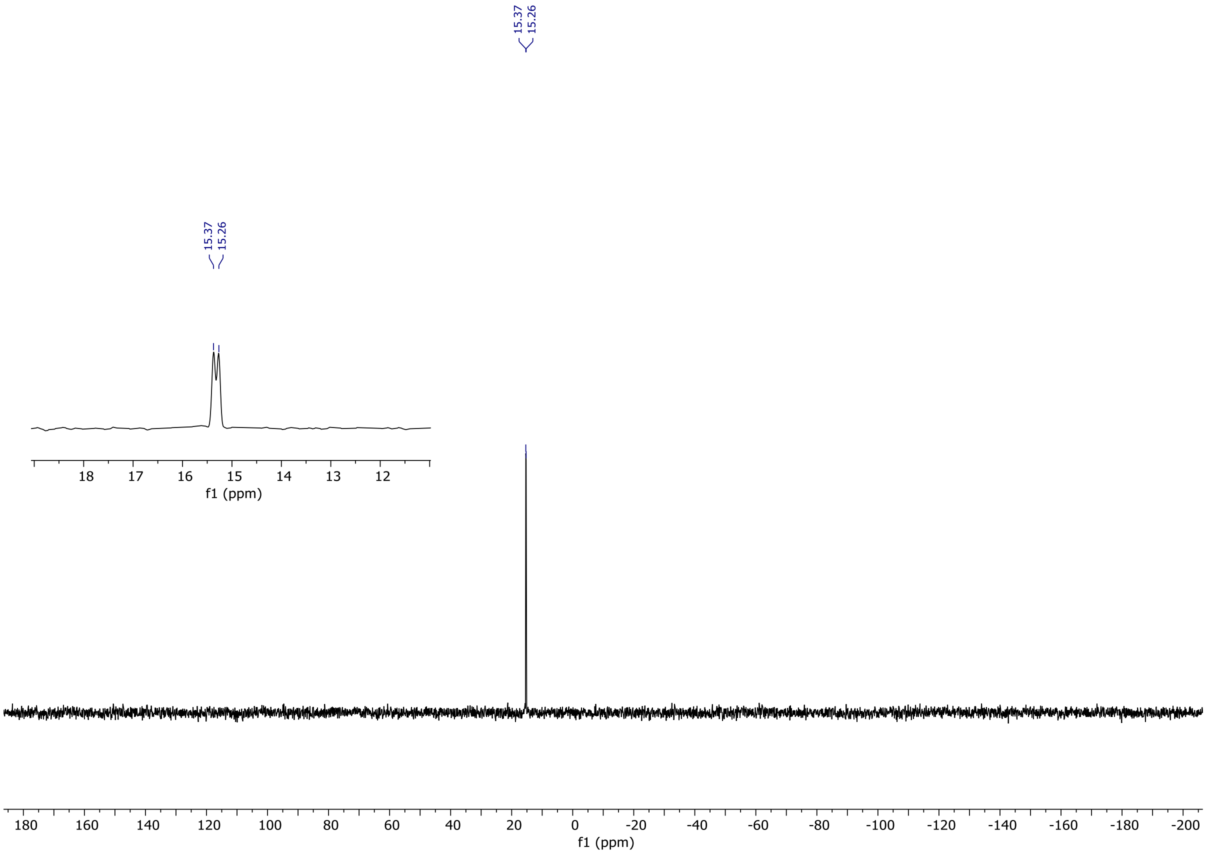

Figure S 5: ^{29}Si NMR of compound **2**.

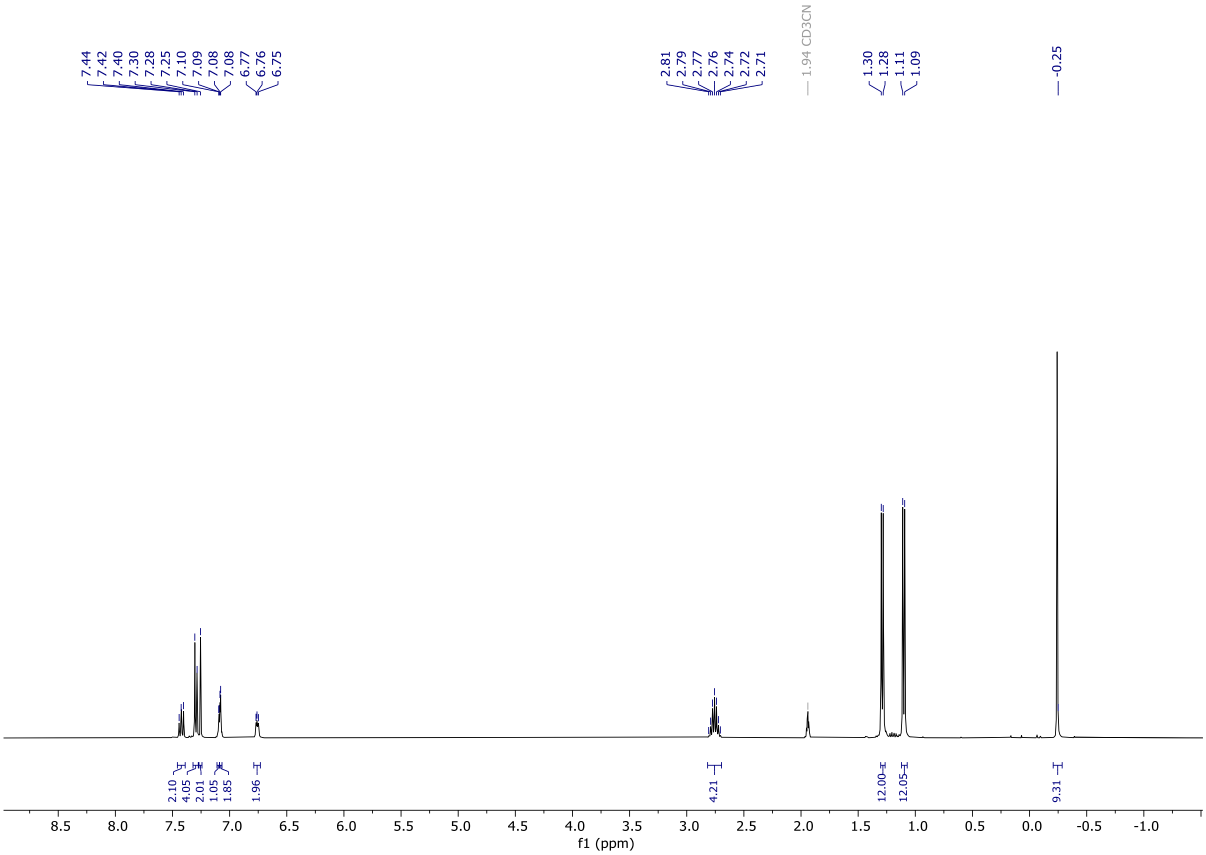

Figure S 6: ^{1}H NMR of compound **3**.

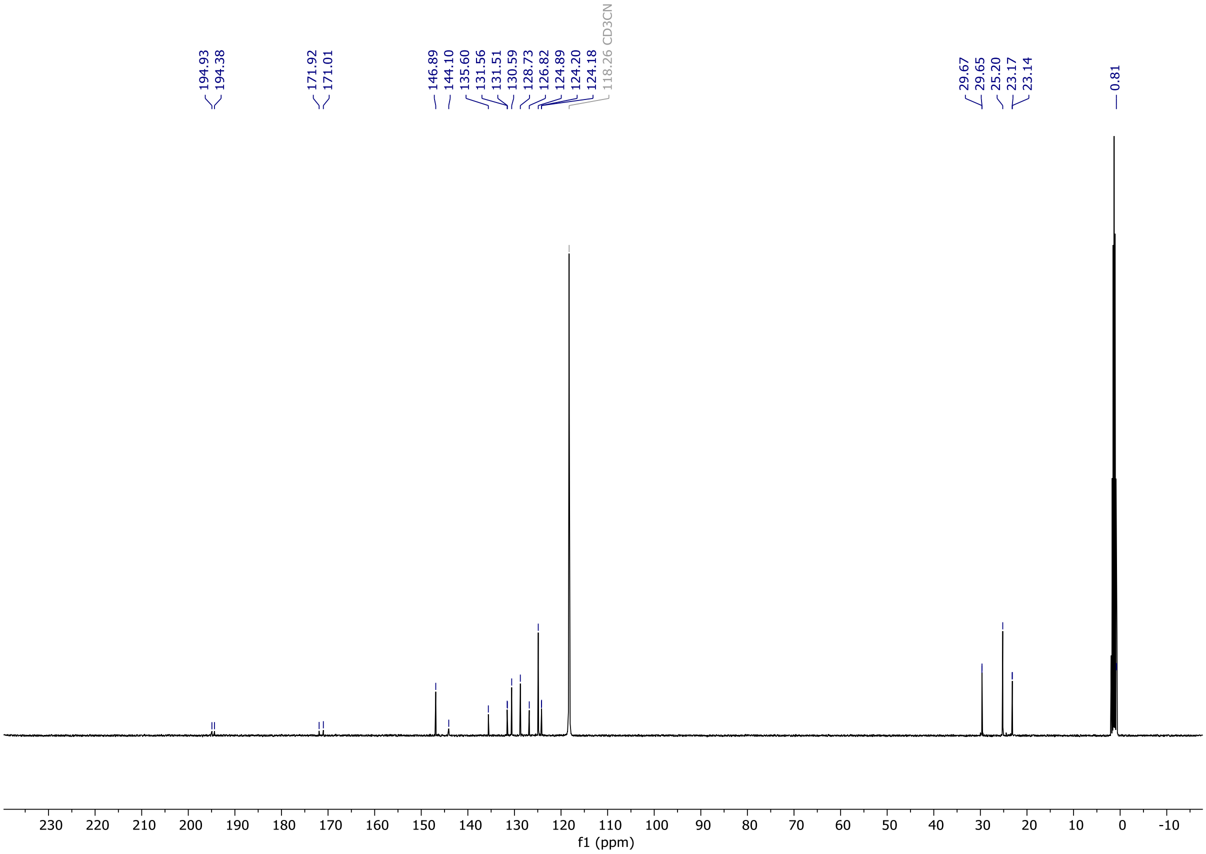

Figure S 7: ^{13}C NMR of compound **3**.

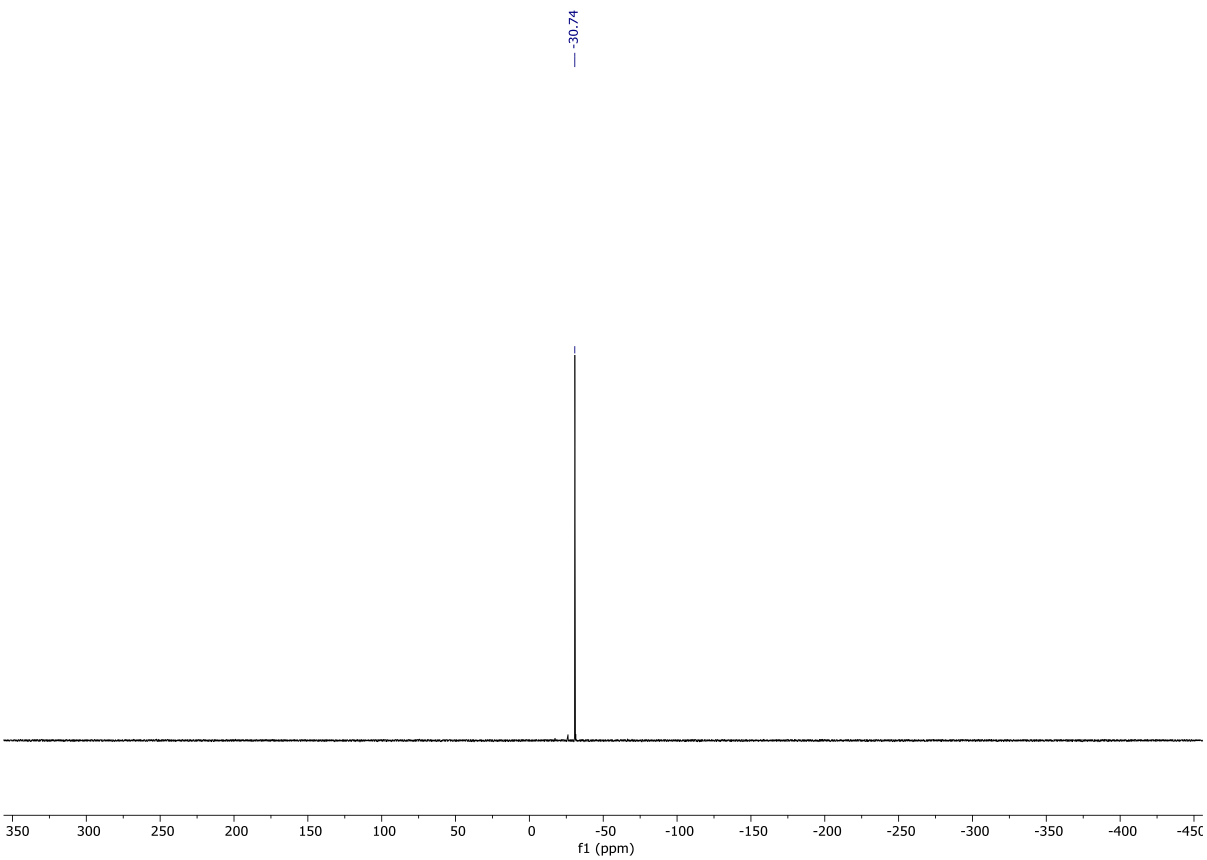

Figure S 8: ^{31}P NMR of compound **3**.

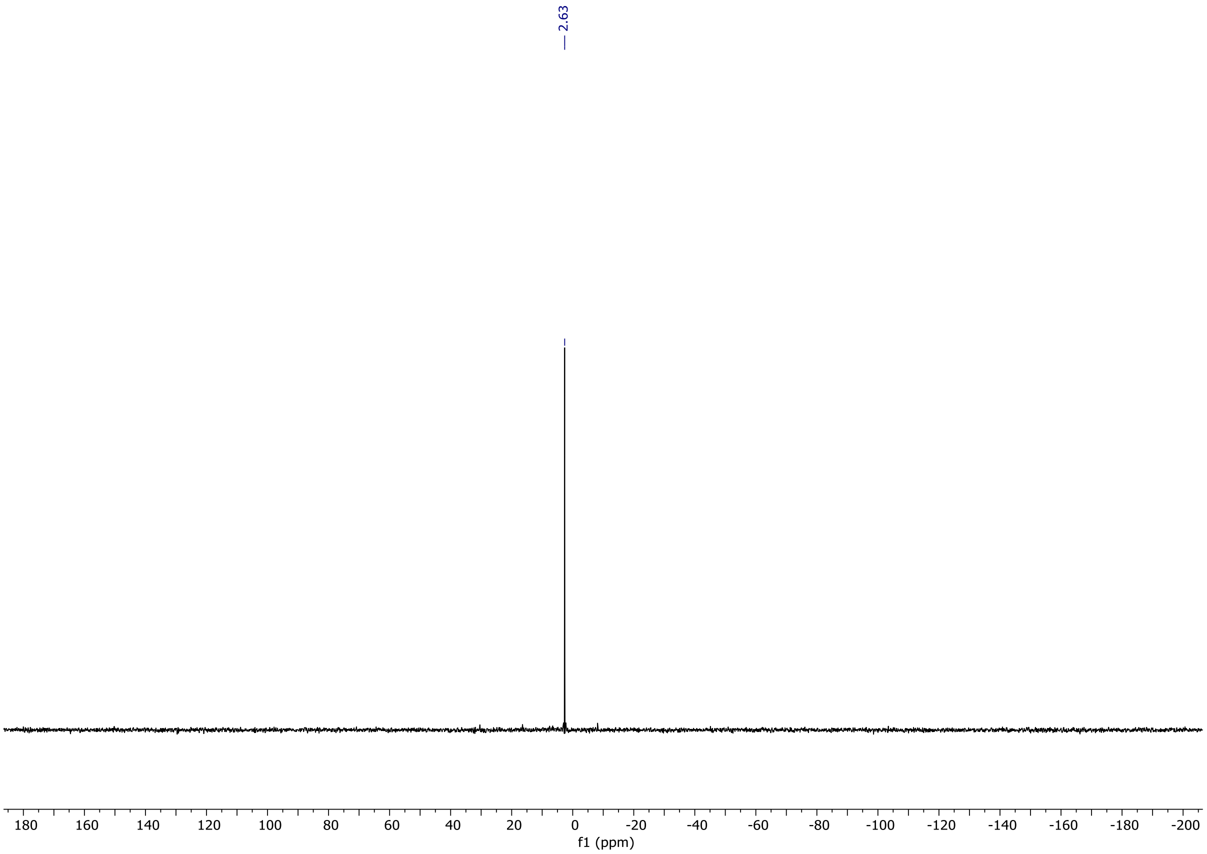

Figure S 9: ^{29}Si NMR of compound **3**.

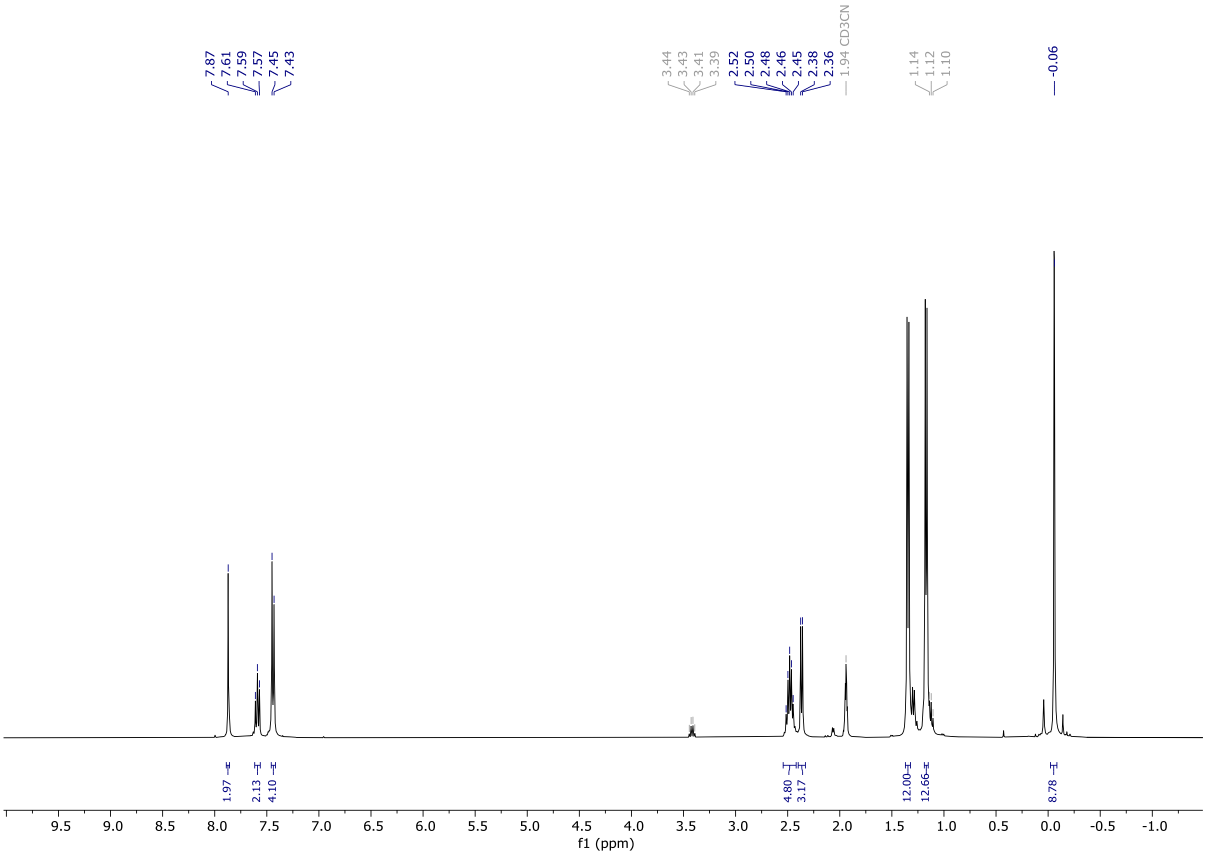

Figure S 10: ^{1}H NMR of compound **4a**. The marked impurities (grey) are Et_2O (3.42, 1.12 ppm).

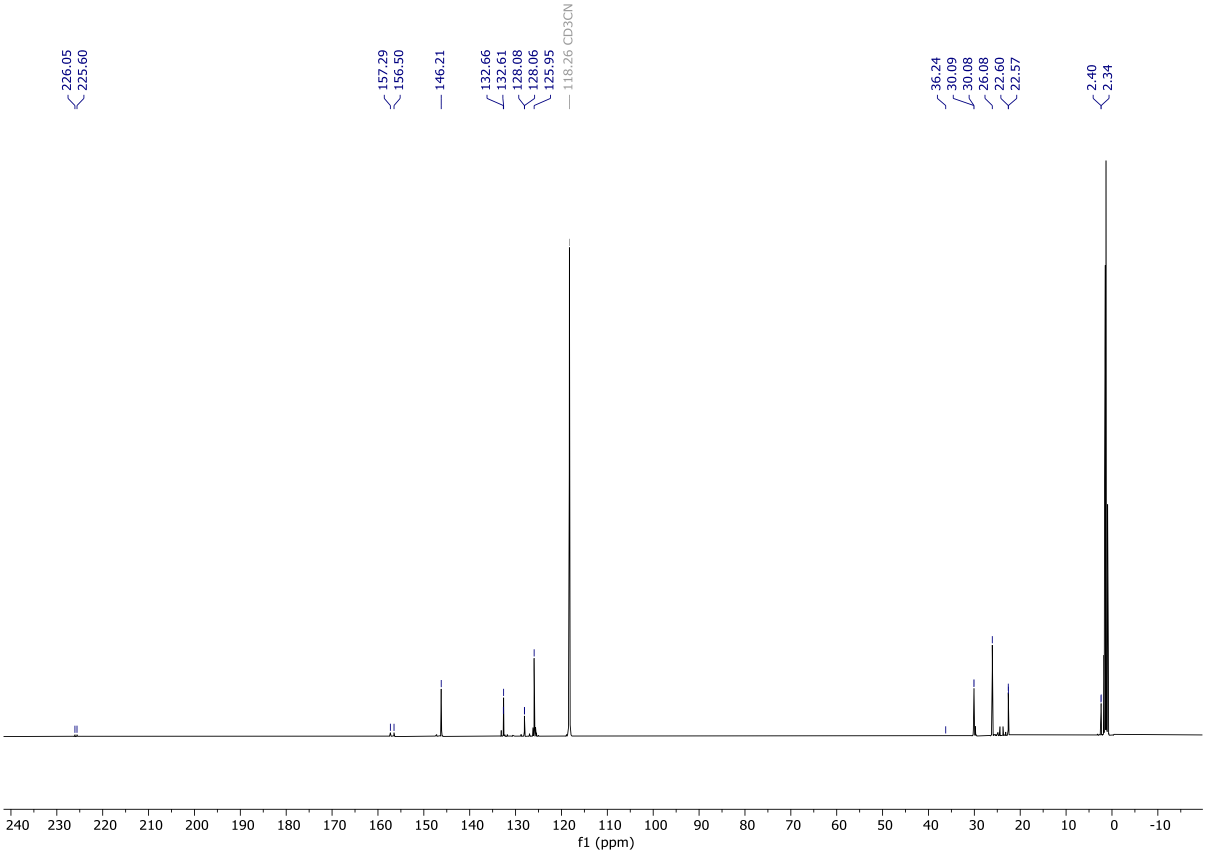

Figure S 11: ^{13}C NMR of compound **4a**.

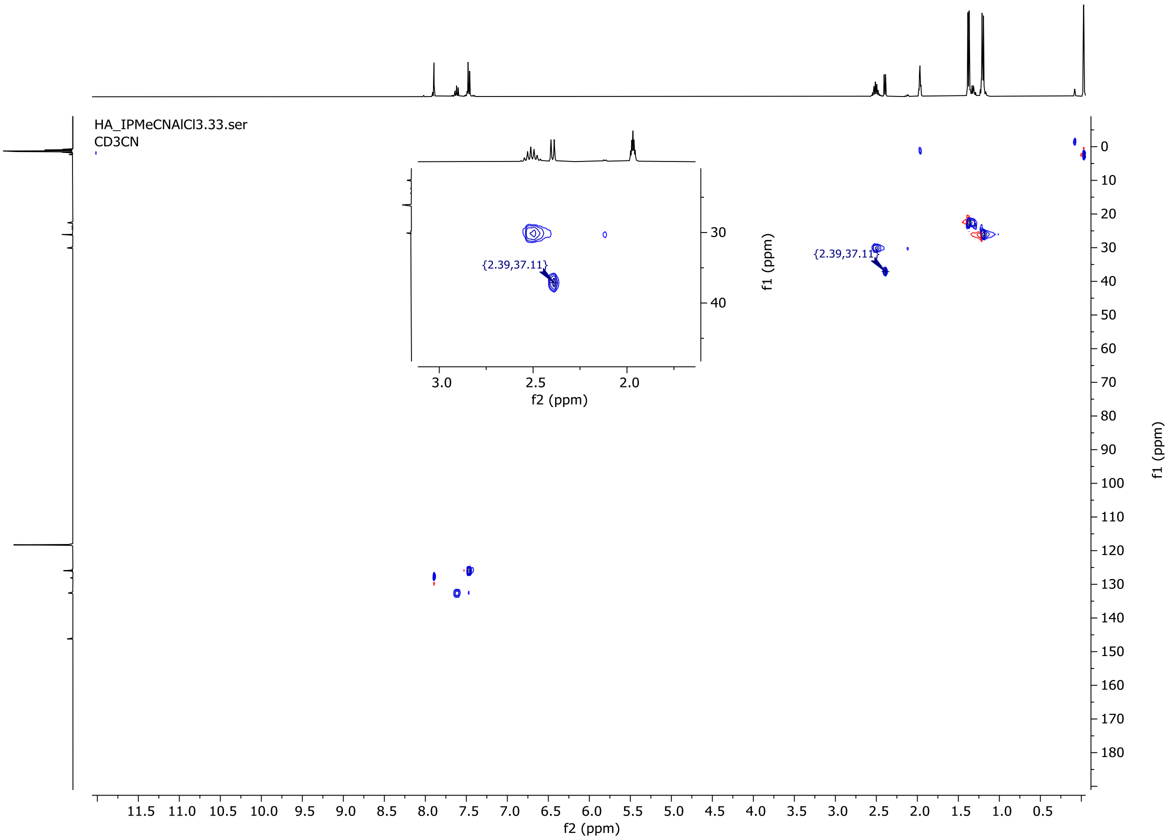

Figure S 12: HSQC of compound **4a**.

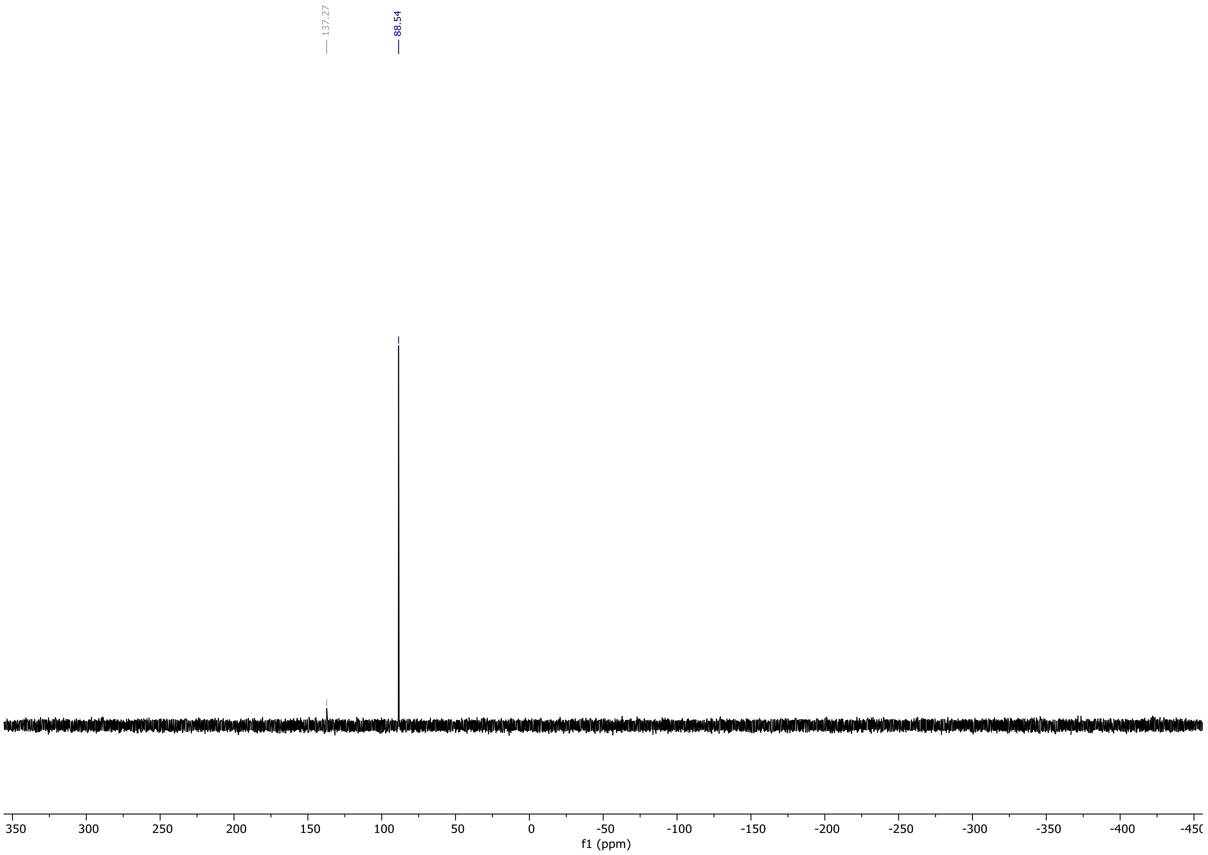

Figure S 13: ^{31}P NMR of compound **4a**. The marked impurities (grey) are **5a**.

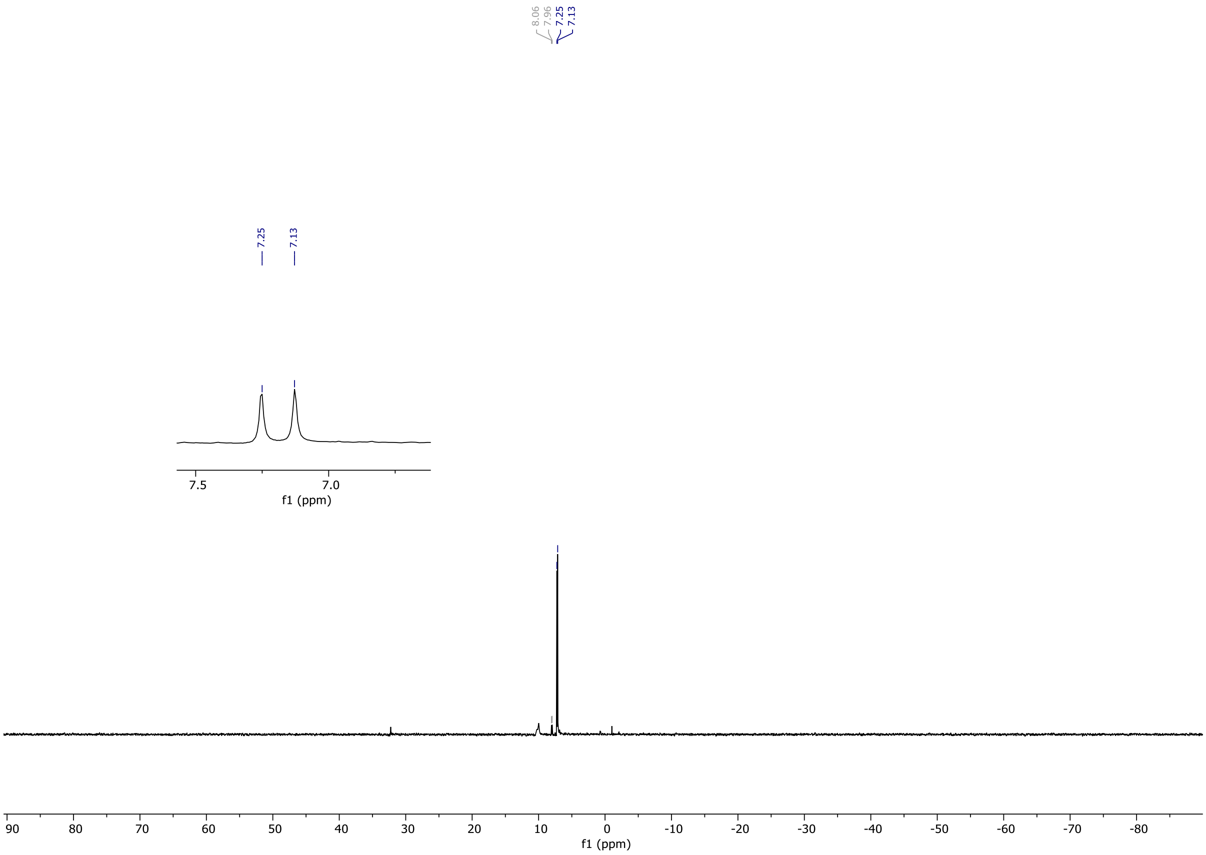

Figure S 14: ^{29}Si NMR of compound **4a**. The marked impurities (grey) are **5a**.

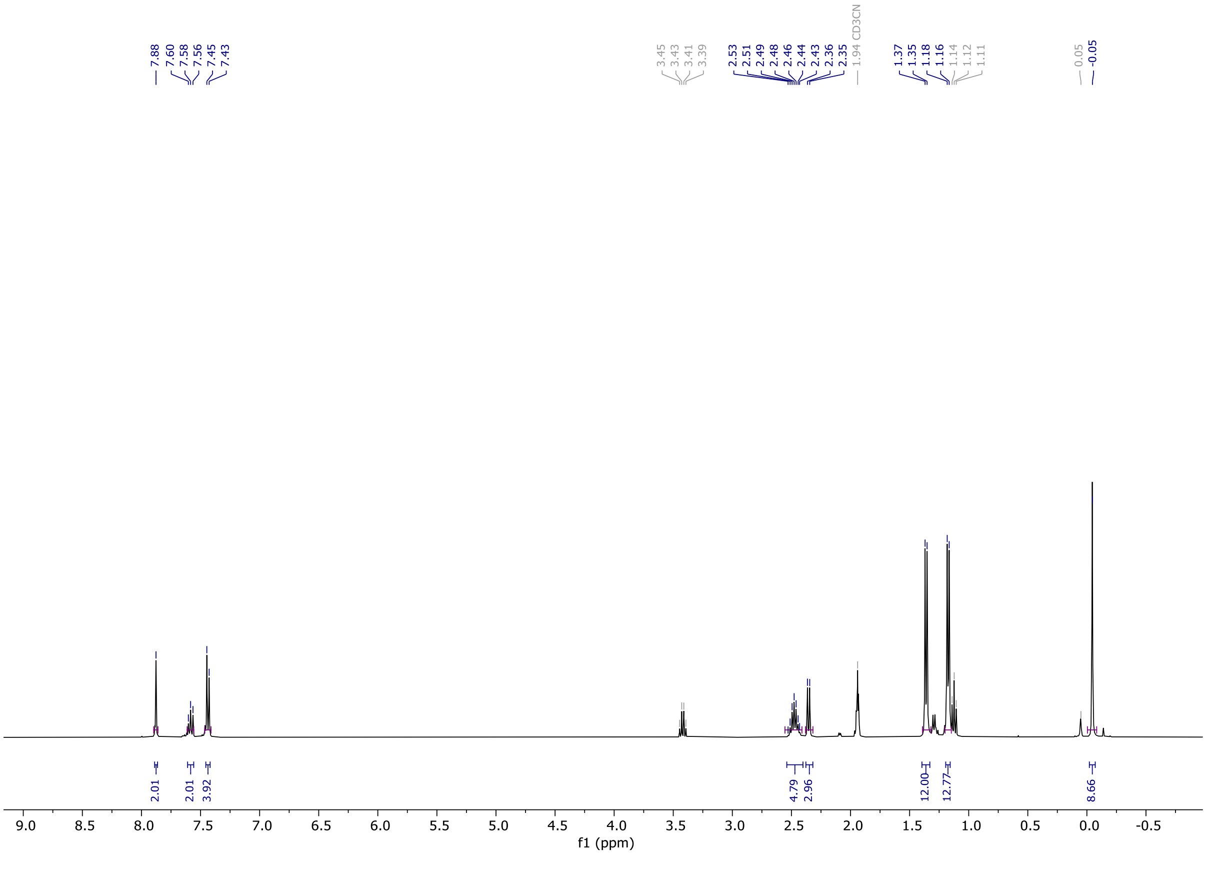

Figure S 15: ^{1}H NMR of compound **4b**. The marked impurities (grey) are Et_2O (3.42, 1.12 ppm).

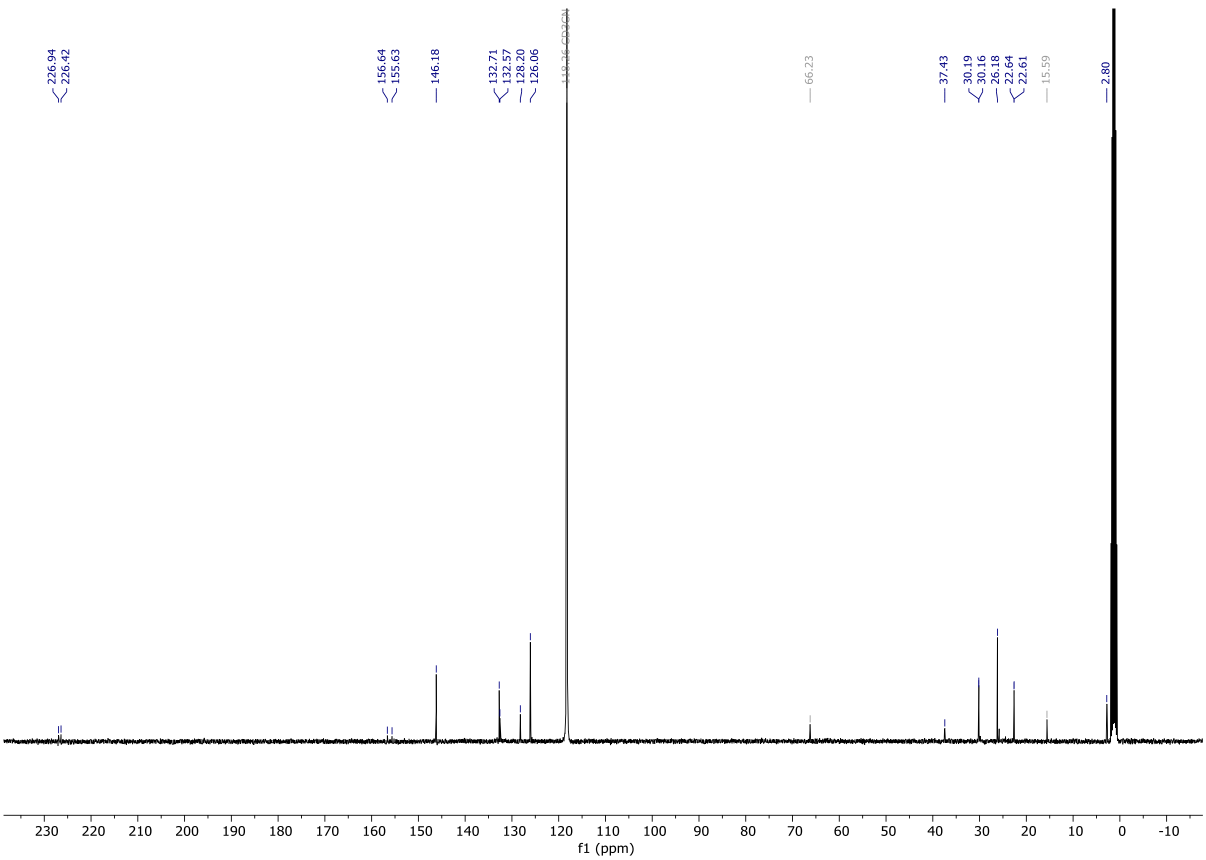

Figure S 16: ^{13}C NMR of compound **4b.** The marked impurities (grey) are Et_2O (66.23, 15.59 ppm).

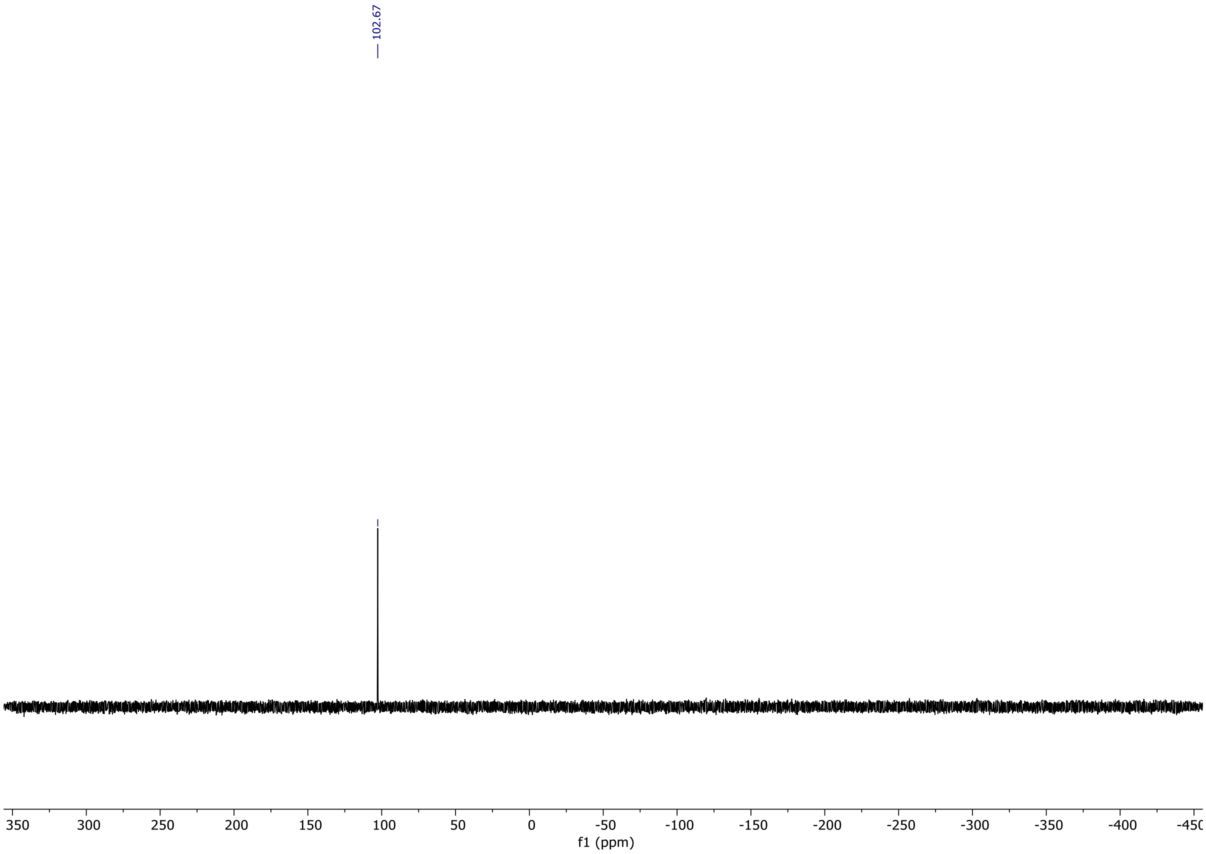

Figure S 17: ^{31}P NMR of compound **4b**.

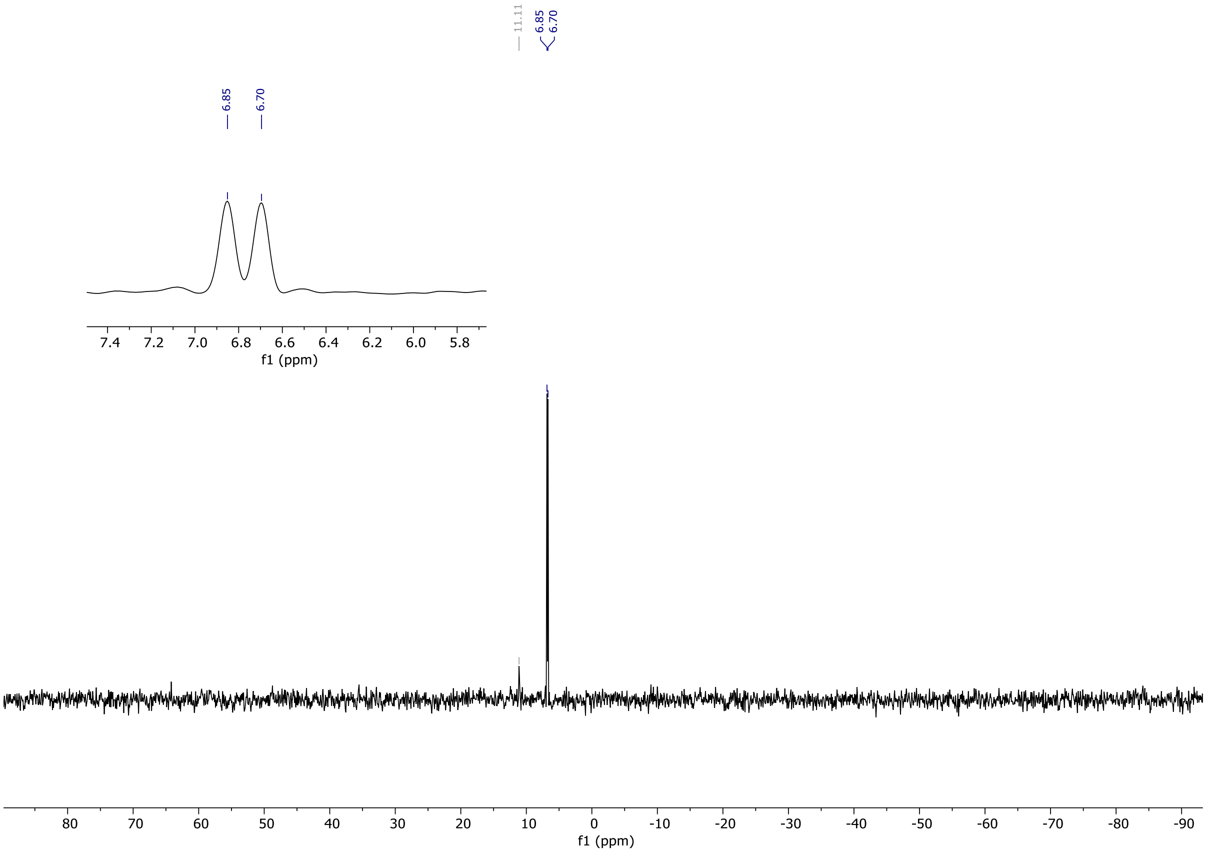

Figure S 18: ^{29}Si NMR of compound **4b**. The marked impurities (grey) are **5b.**

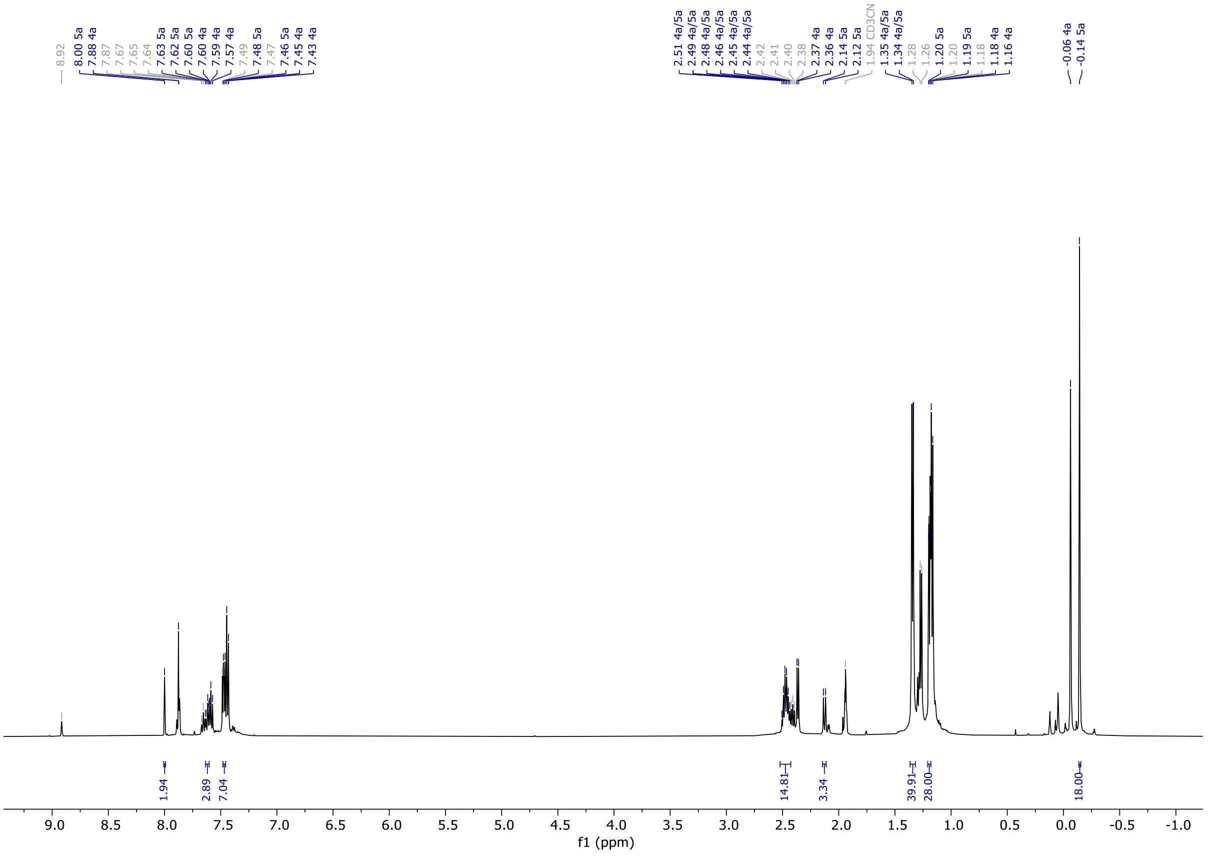

Figure S 19: ^{1}H NMR of compound **5a**. The marked impurities (grey) are Imidazolium salt.

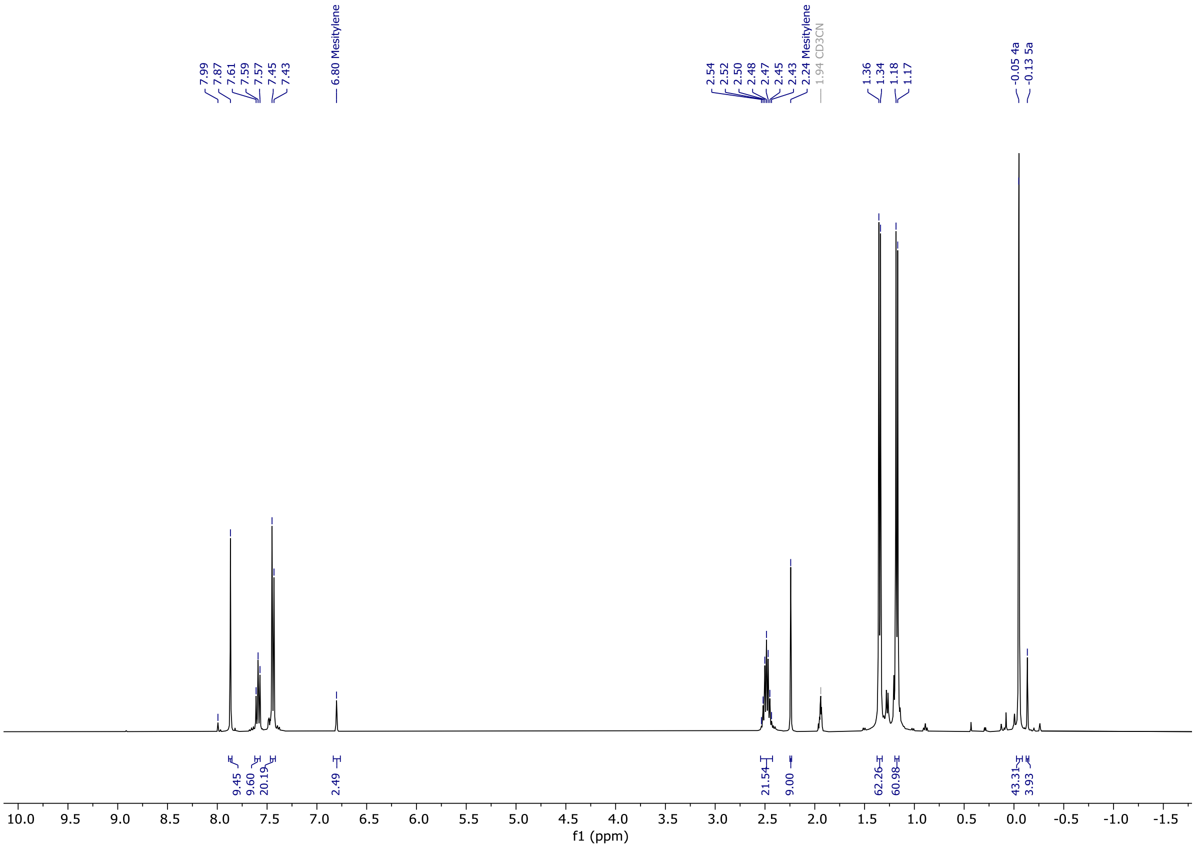

Figure S 20: ^{1}H NMR of reaction solution of **5a** with mesitylene as internal standard.

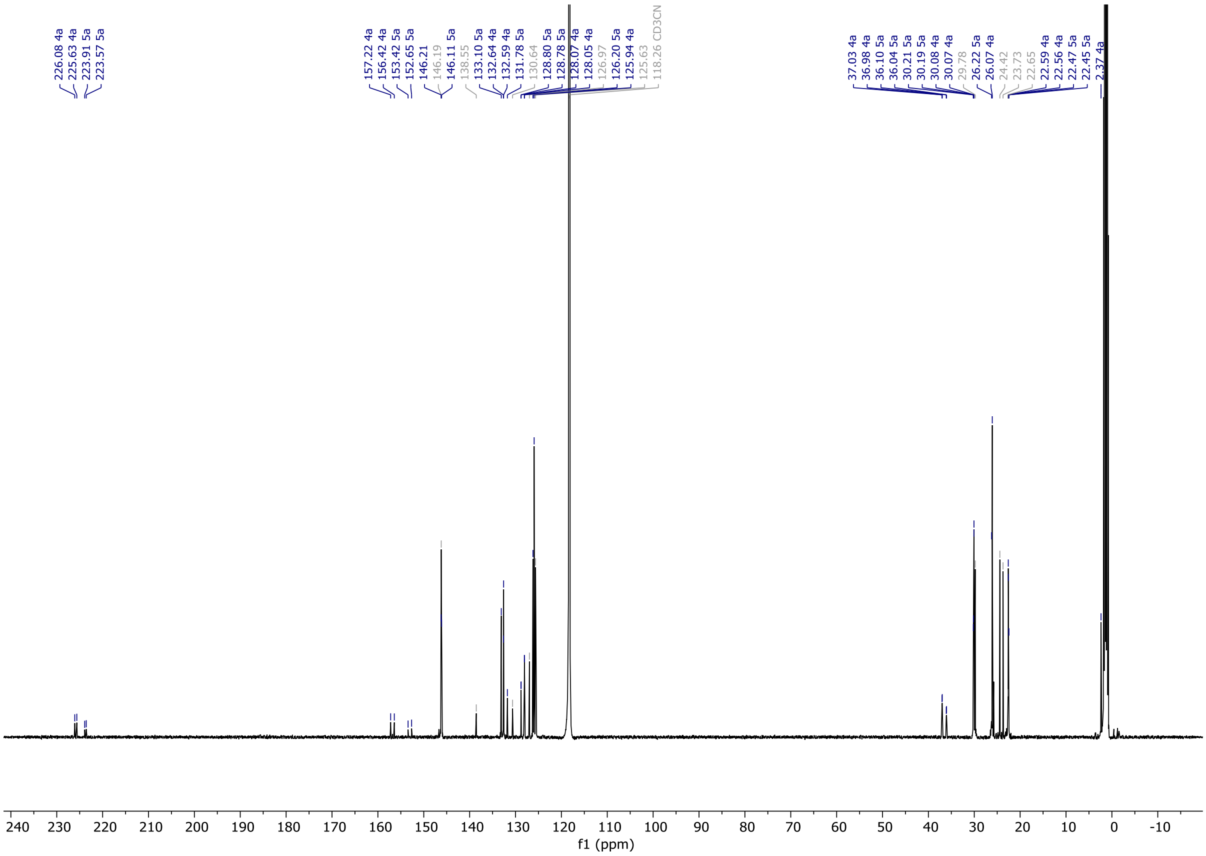

Figure S 21: ^{13}C NMR of compound **5a**. The marked impurities (grey) are Imidazolium salt.

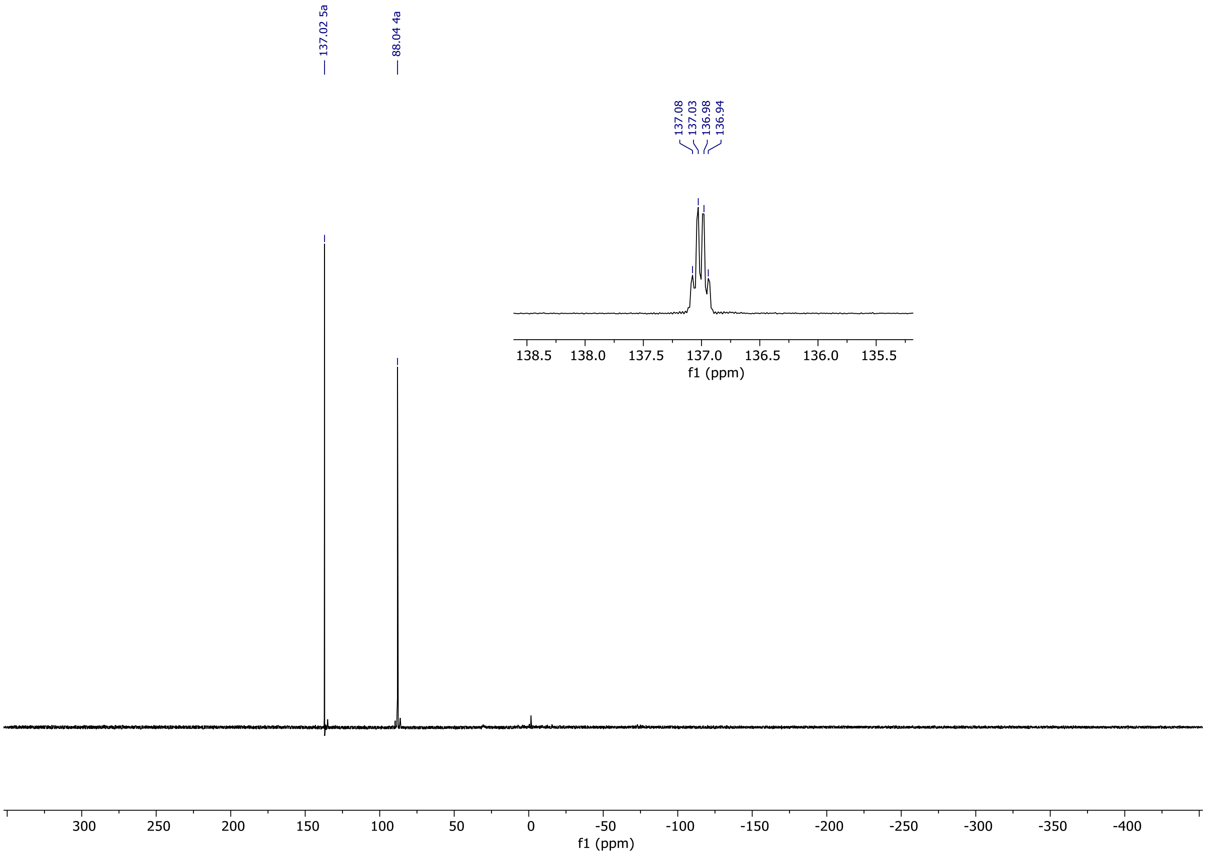

Figure S 22: ^{31}P NMR of compound **5a**. Note: The zoomed spectrum was measured separately using a narrower spectral range and a higher number of scans.

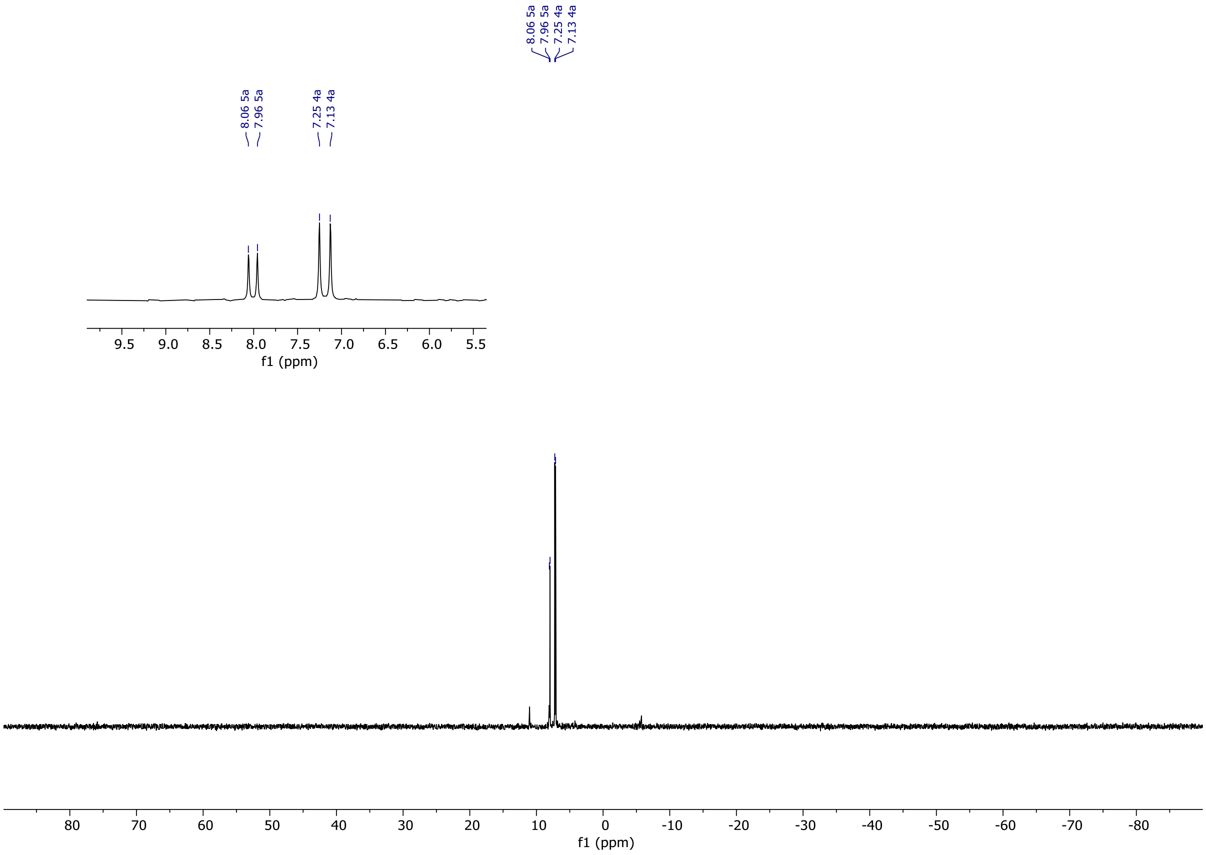

Figure S 23: ^{29}Si NMR of compound **5a**.

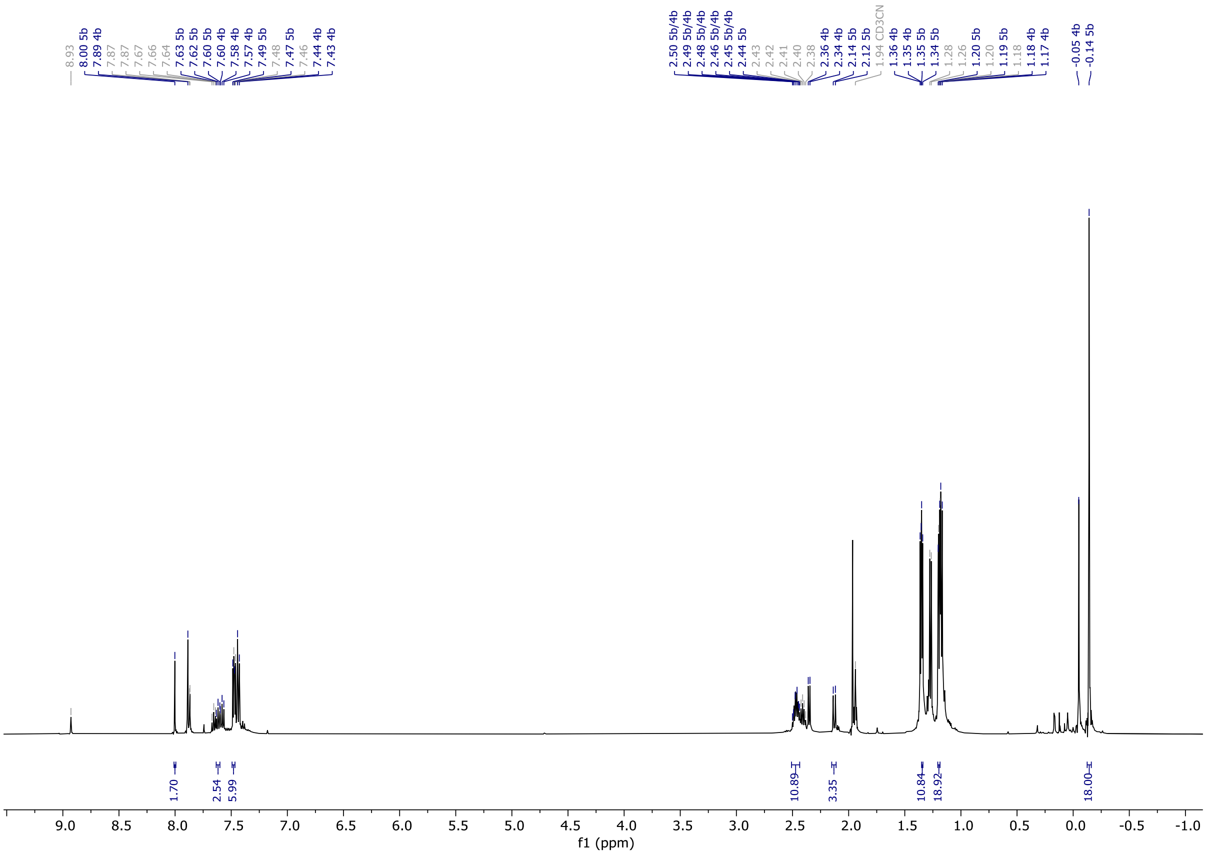

Figure S 24: ^{1}H NMR of compound **5b**. The marked impurities (grey) are Imidazolium salt.

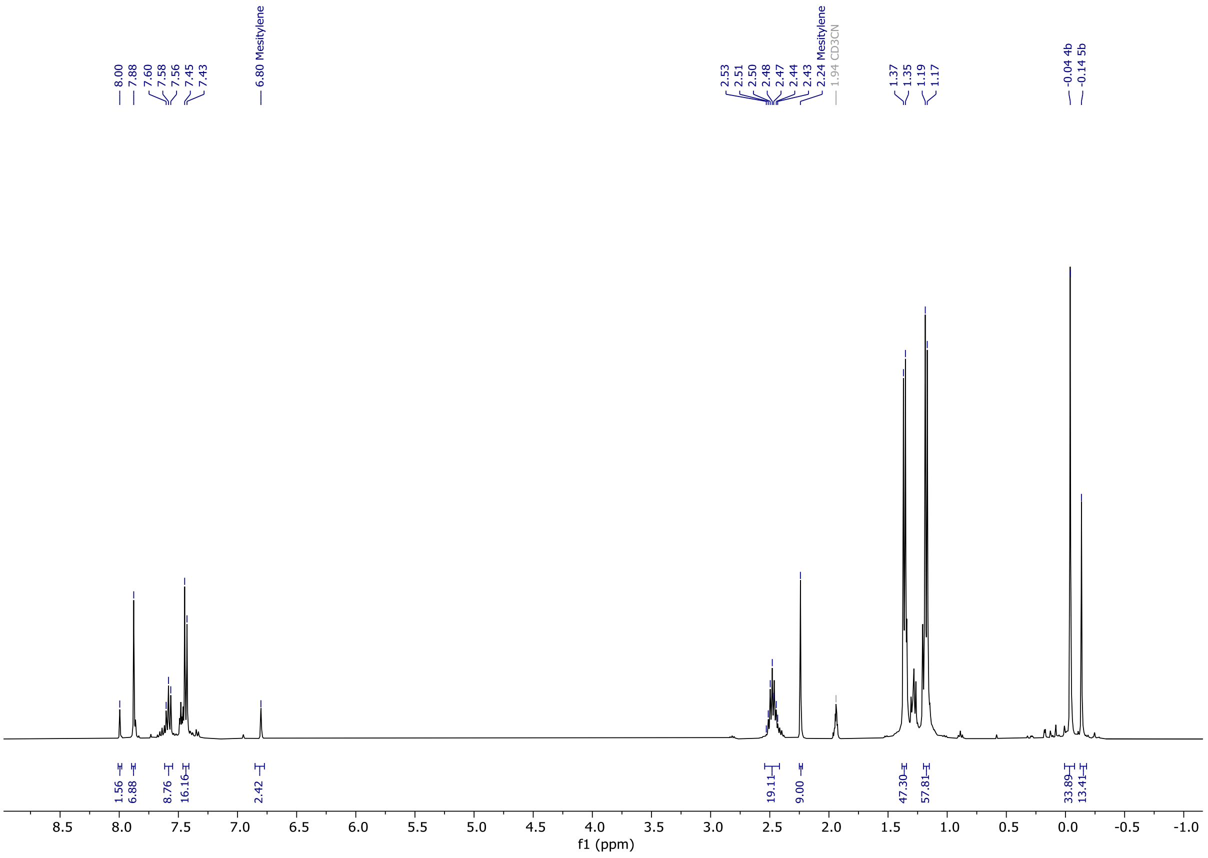

Figure S 25: ^{1}H NMR of reaction solution of **5b** with mesitylene as internal standard.

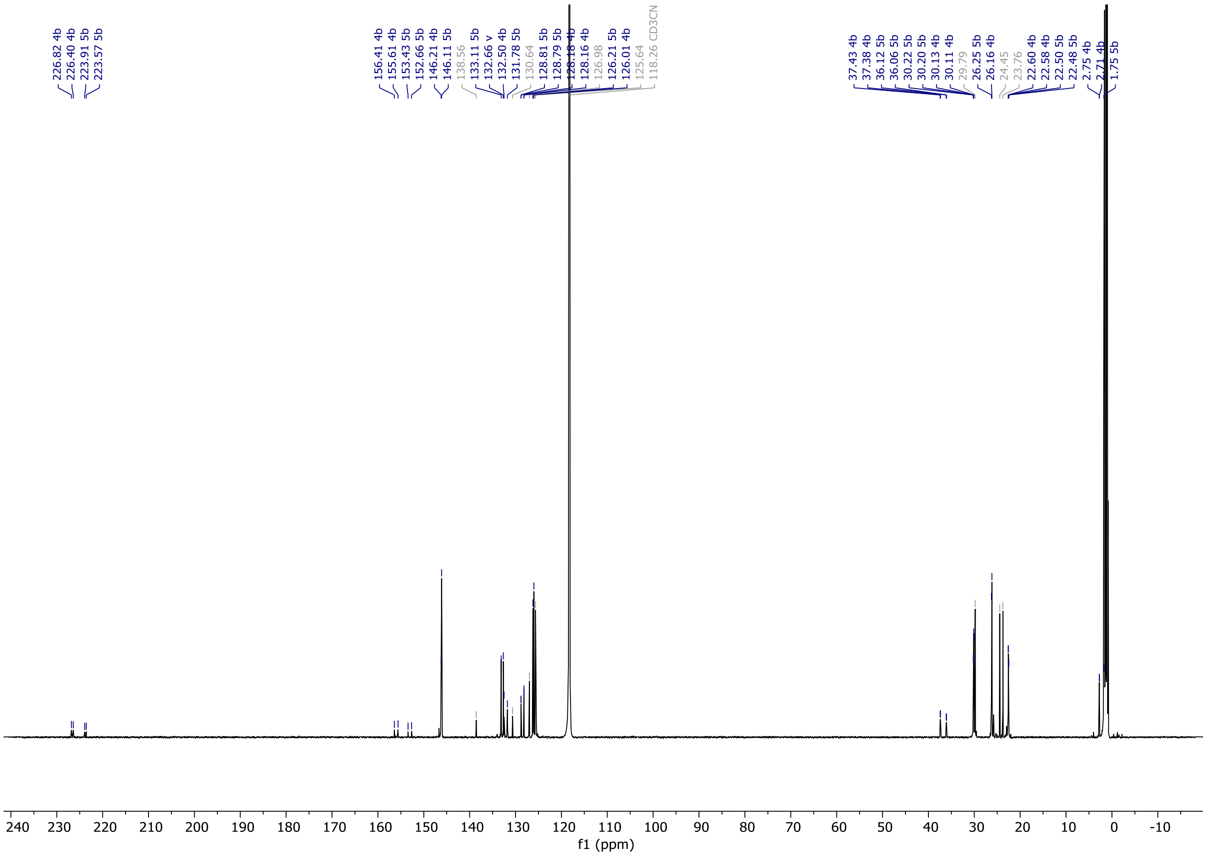

Figure S 26: ^{13}C NMR of compound **5b**. The marked impurities (grey) are Imidazolium salt.

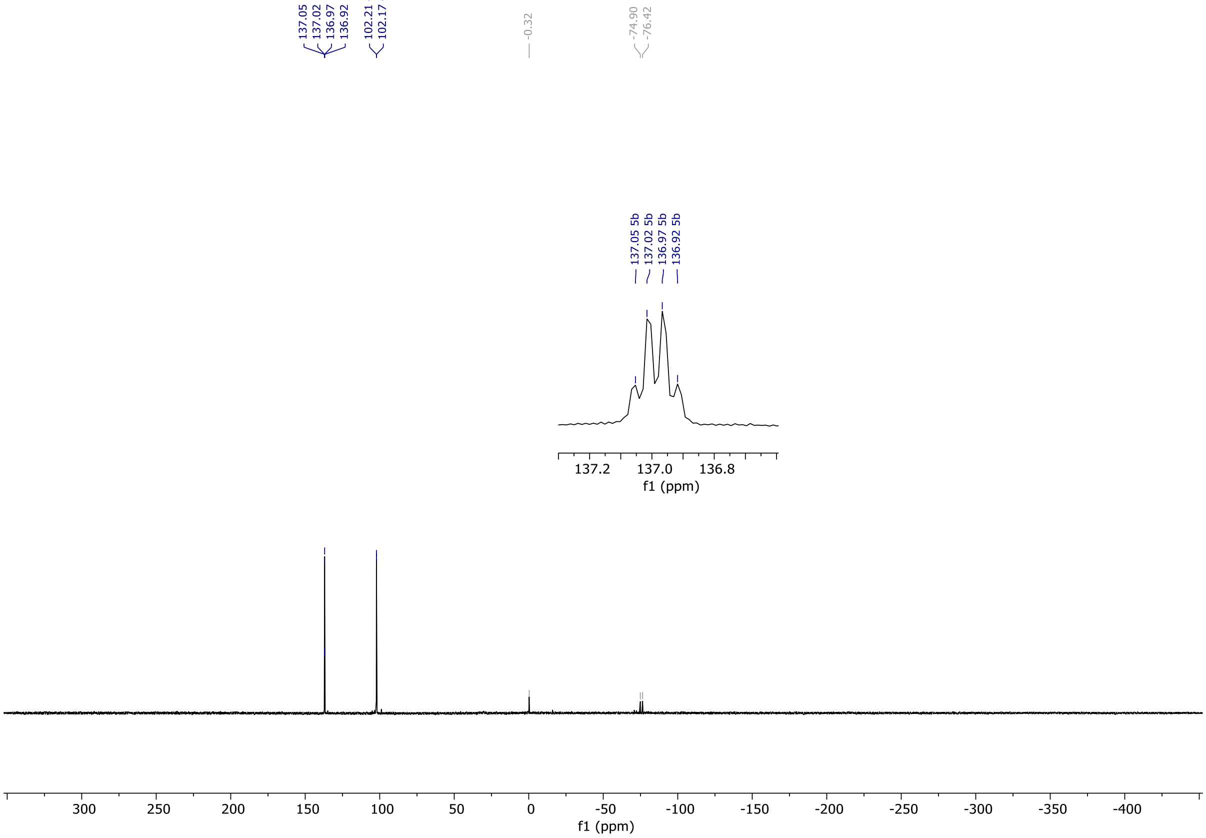

Figure S 27: ^{31}P NMR of compound **5b.**

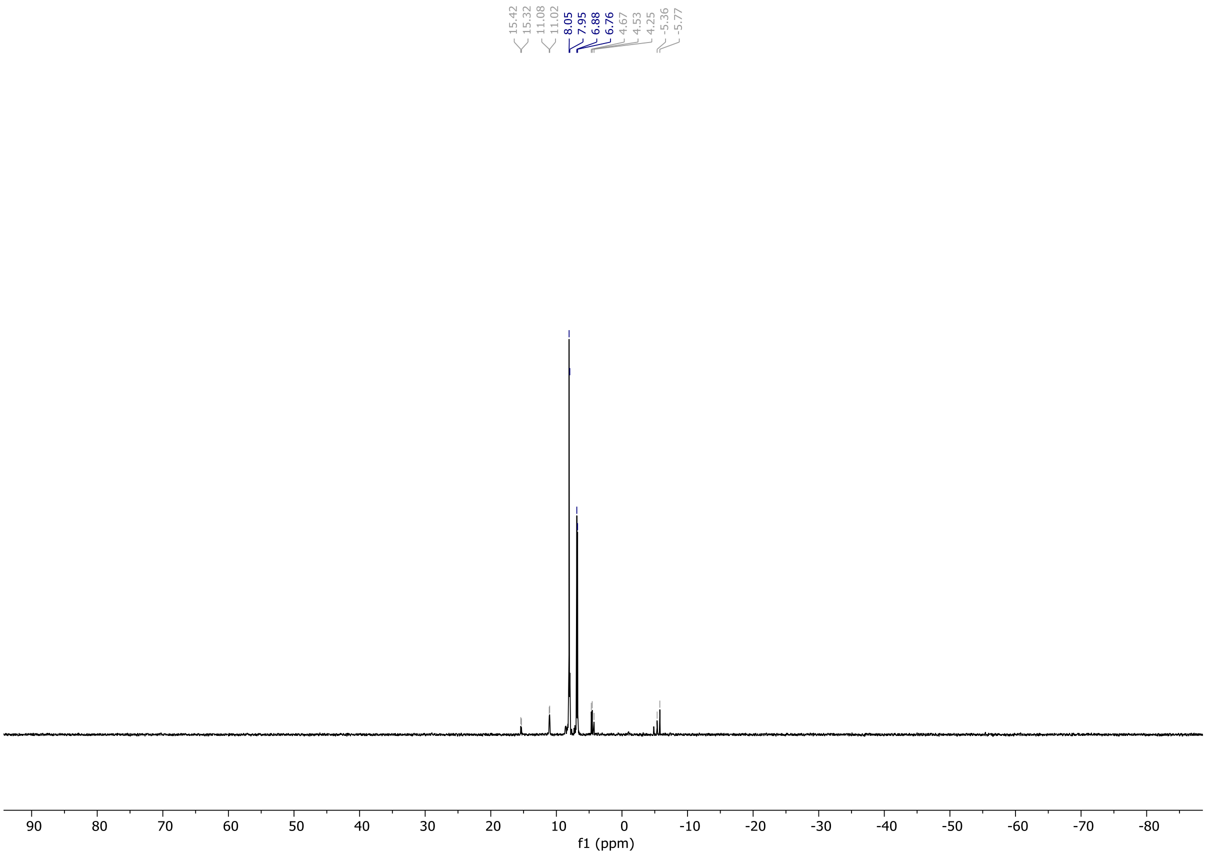

Figure S 28:^{29}Si NMR of compound **5b**.

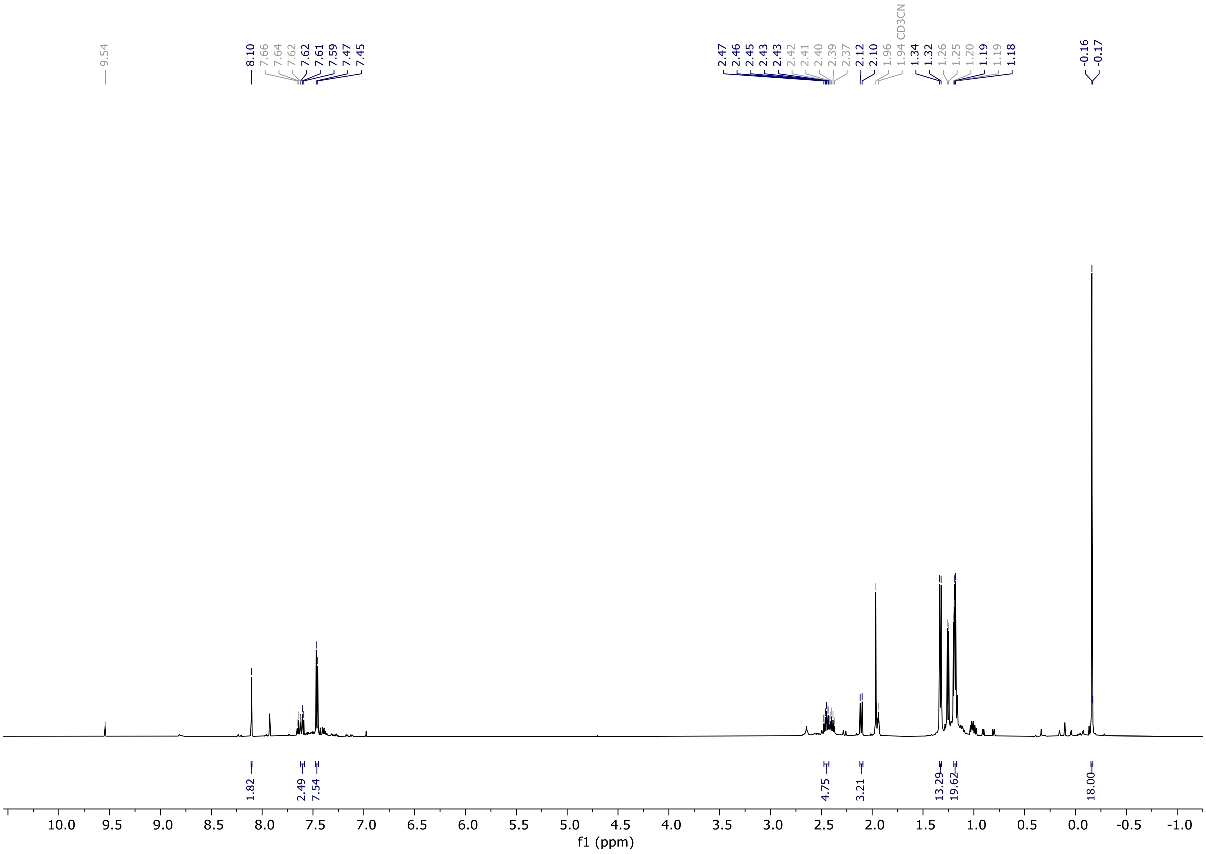

Figure S 29: ^{1}H NMR of compound **5c**. The marked impurities (grey) are Imidazolium salt.

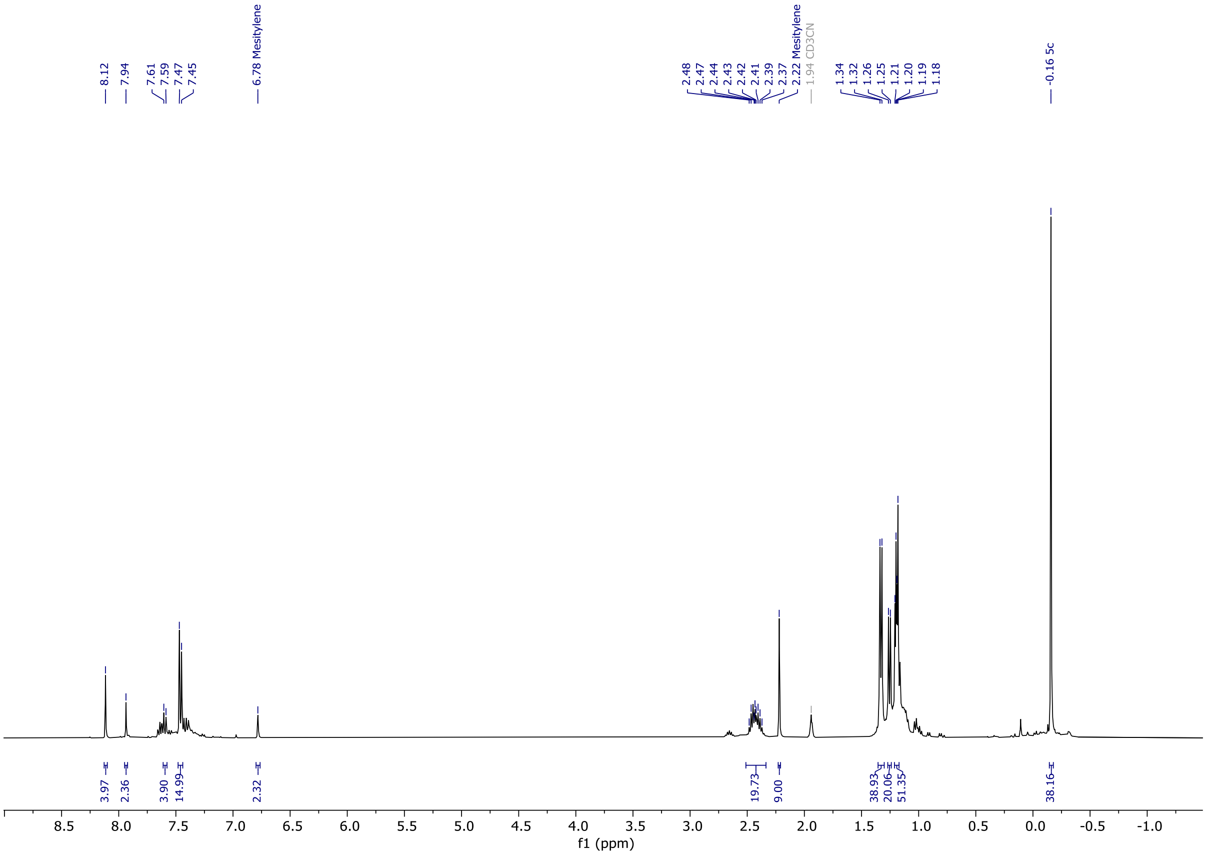

Figure S 30: ^{1}H NMR of reaction solution of **5c** with mesitylene as internal standard.

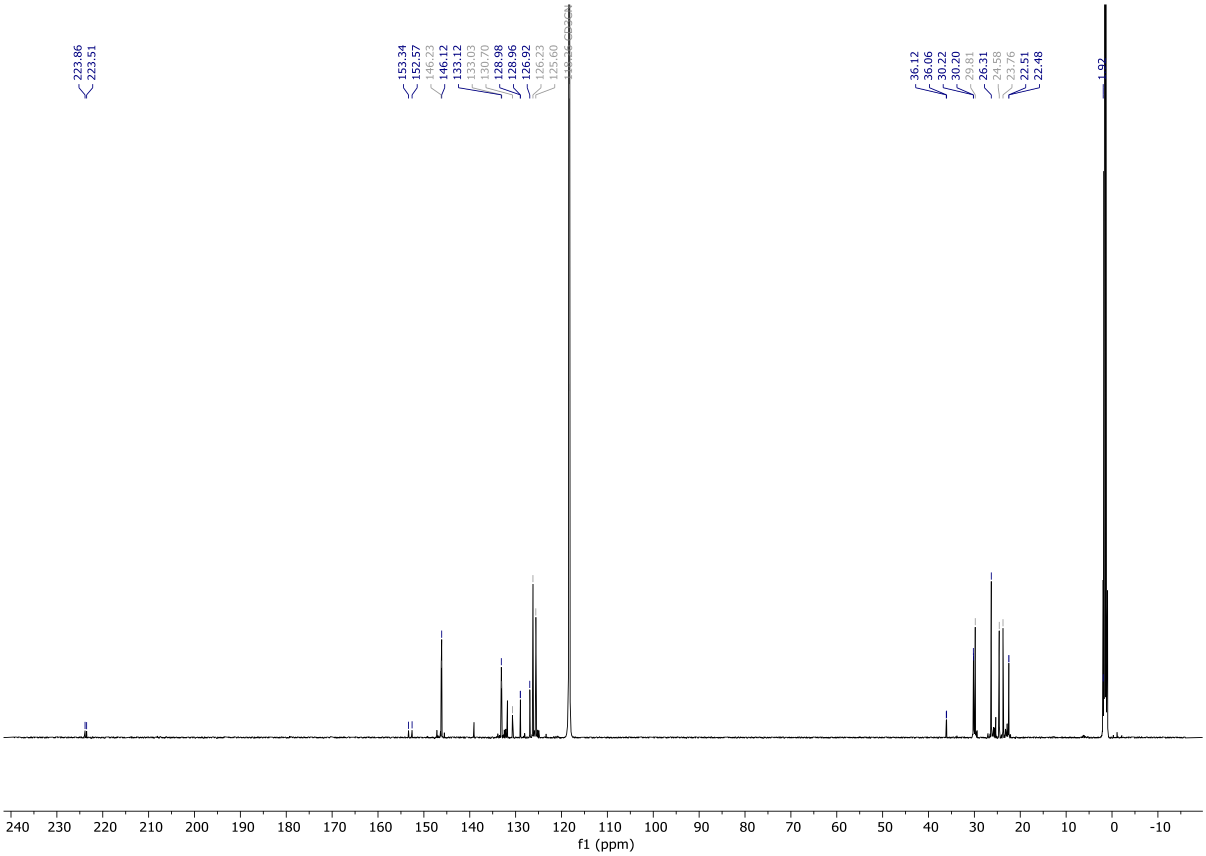

Figure S 31: ^{13}C NMR of compound **5c**. The marked impurities (grey) are Imidazolium salt.

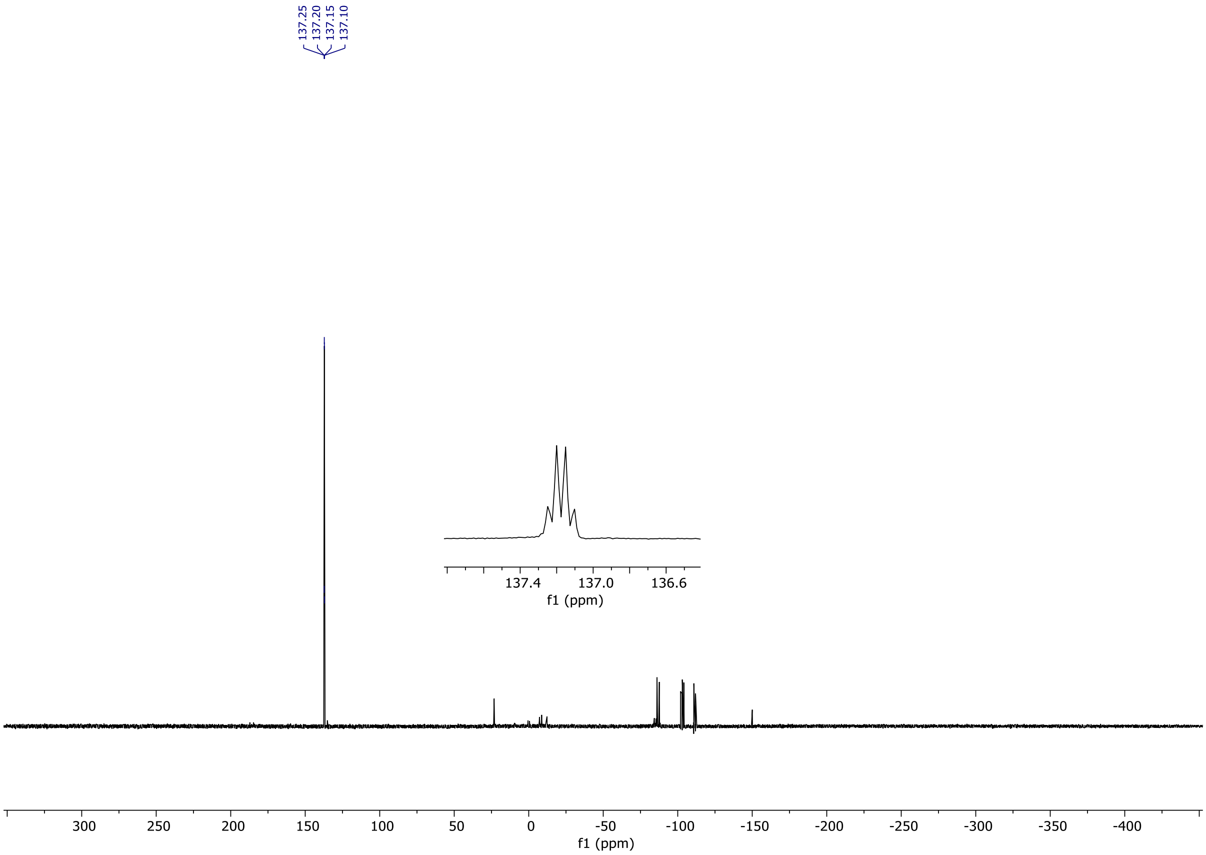

Figure S 32: ^{31}P NMR of compound **5c.**

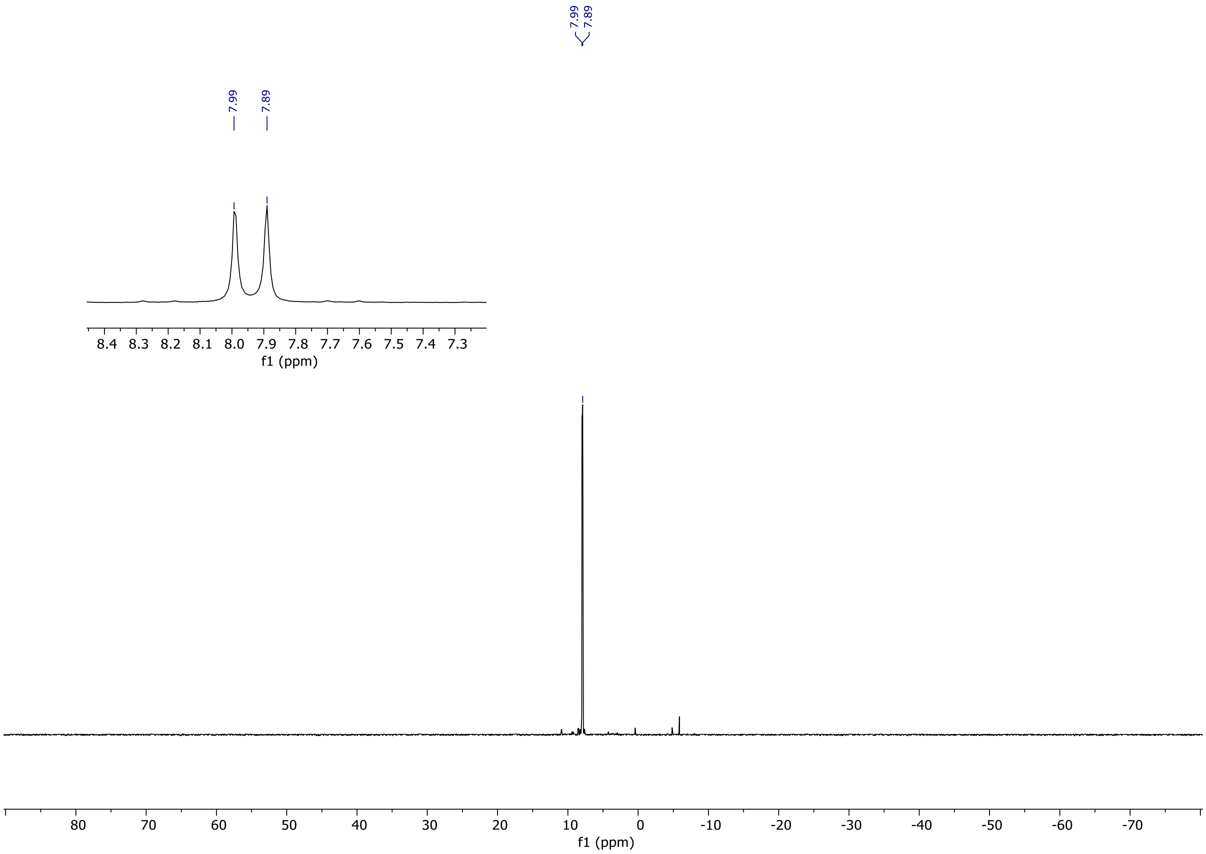

Figure S 33: ^{29}Si NMR of compound **5c**.

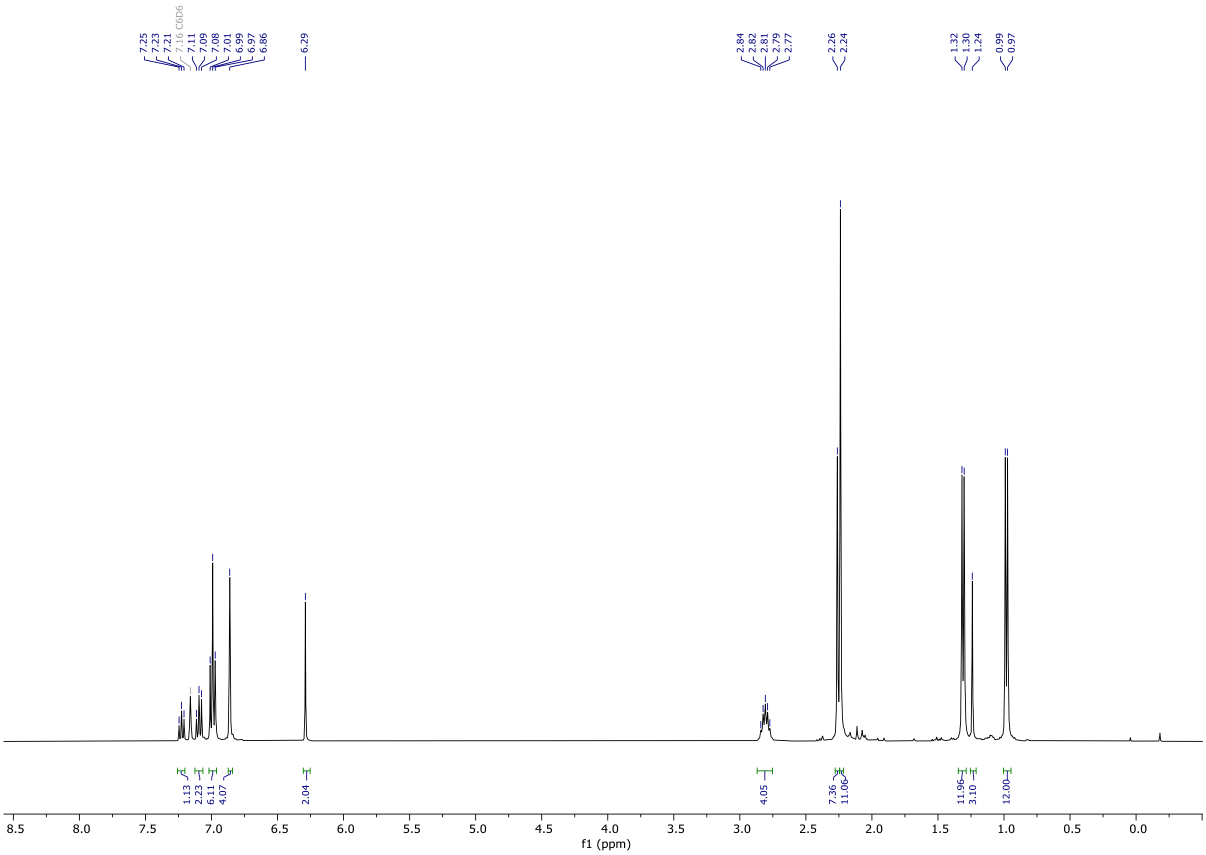

Figure S 34: ^{1}H NMR of compound **6.**

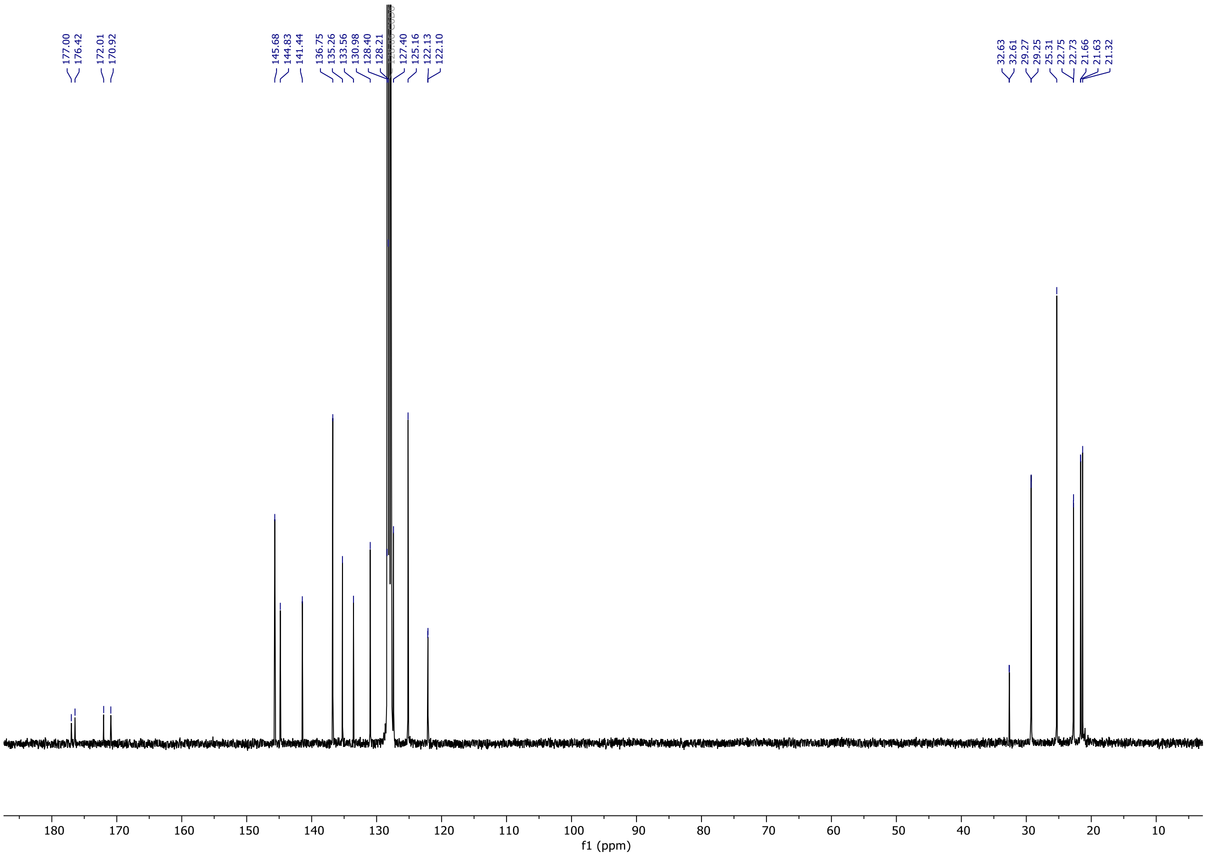

Figure S 35: 13C NMR of compound **6.**

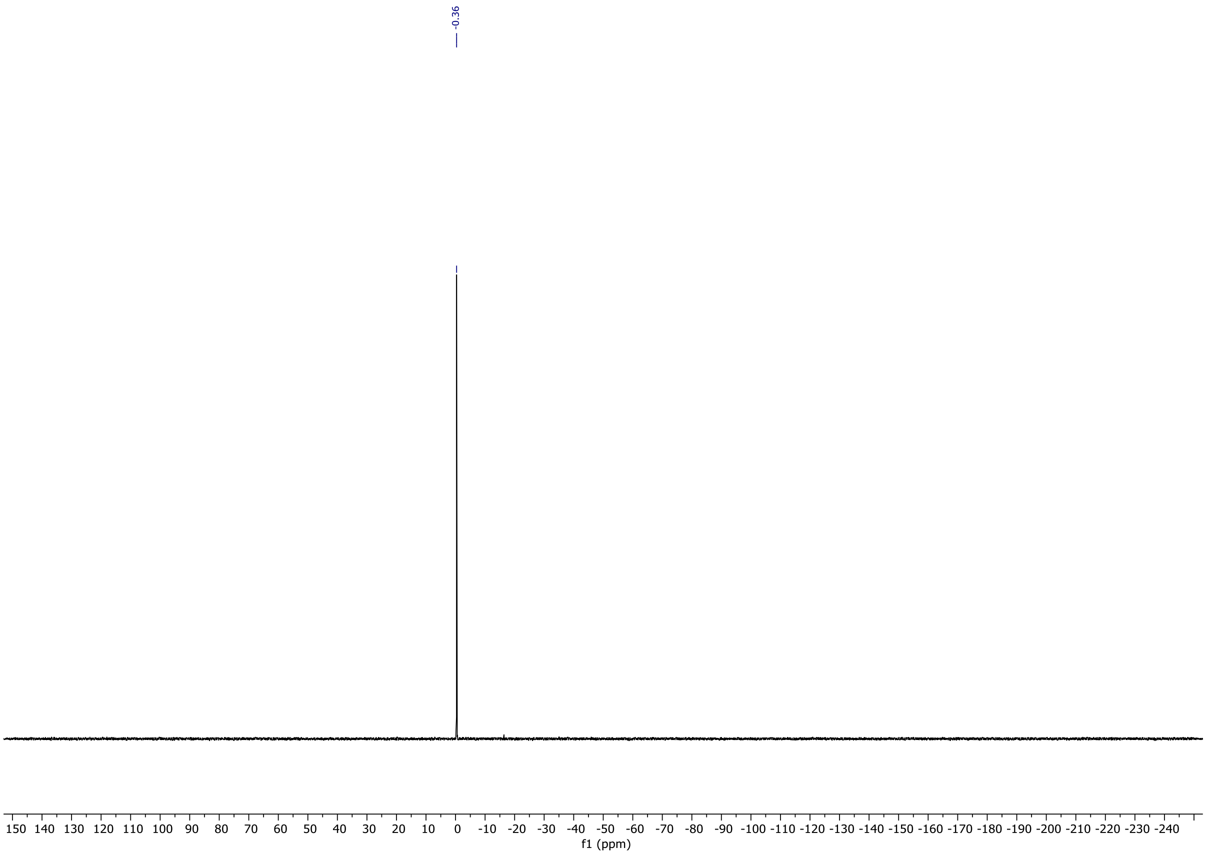

Figure S 36: ^{31}P NMR of compound **6.**

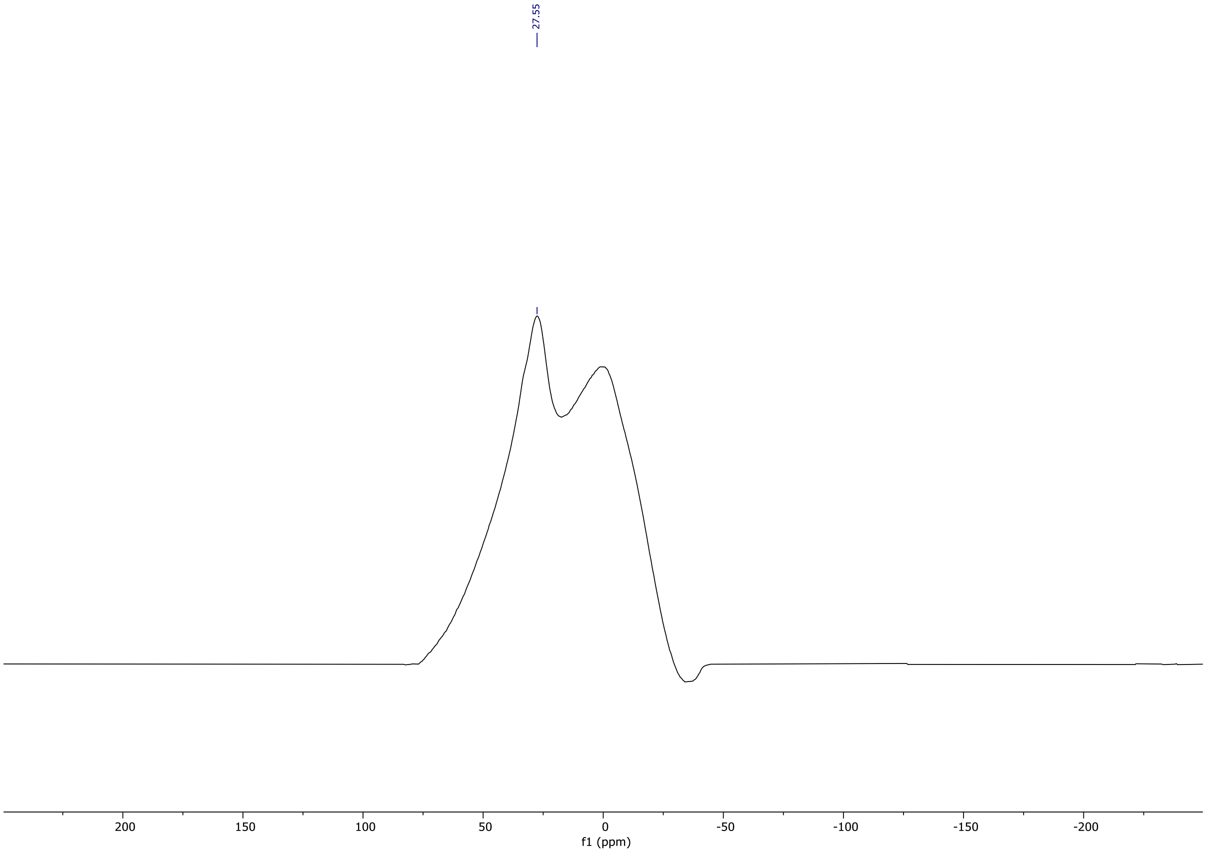

Figure S 37: ^{11}B NMR of compound **6.**

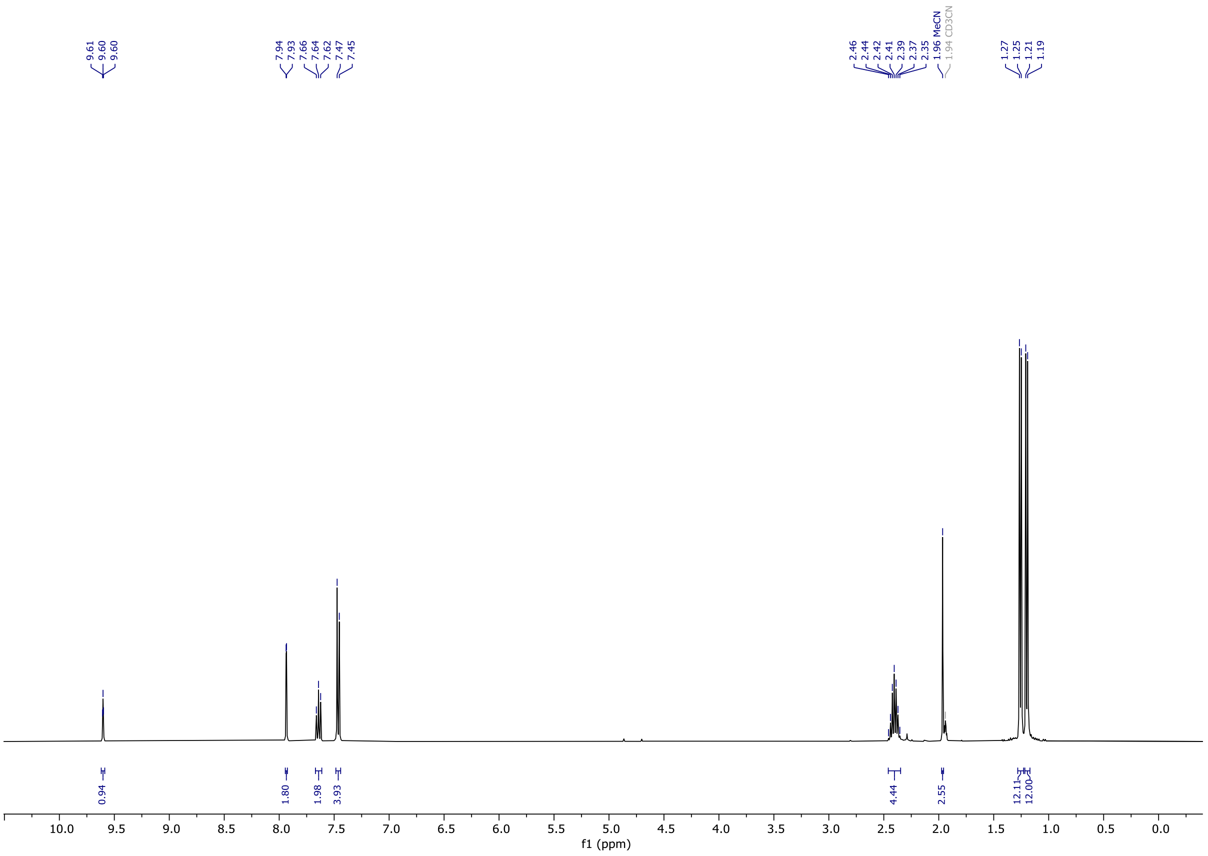

Figure S 38: ^{1}H NMR of compound **IDippAlI$_4$.**

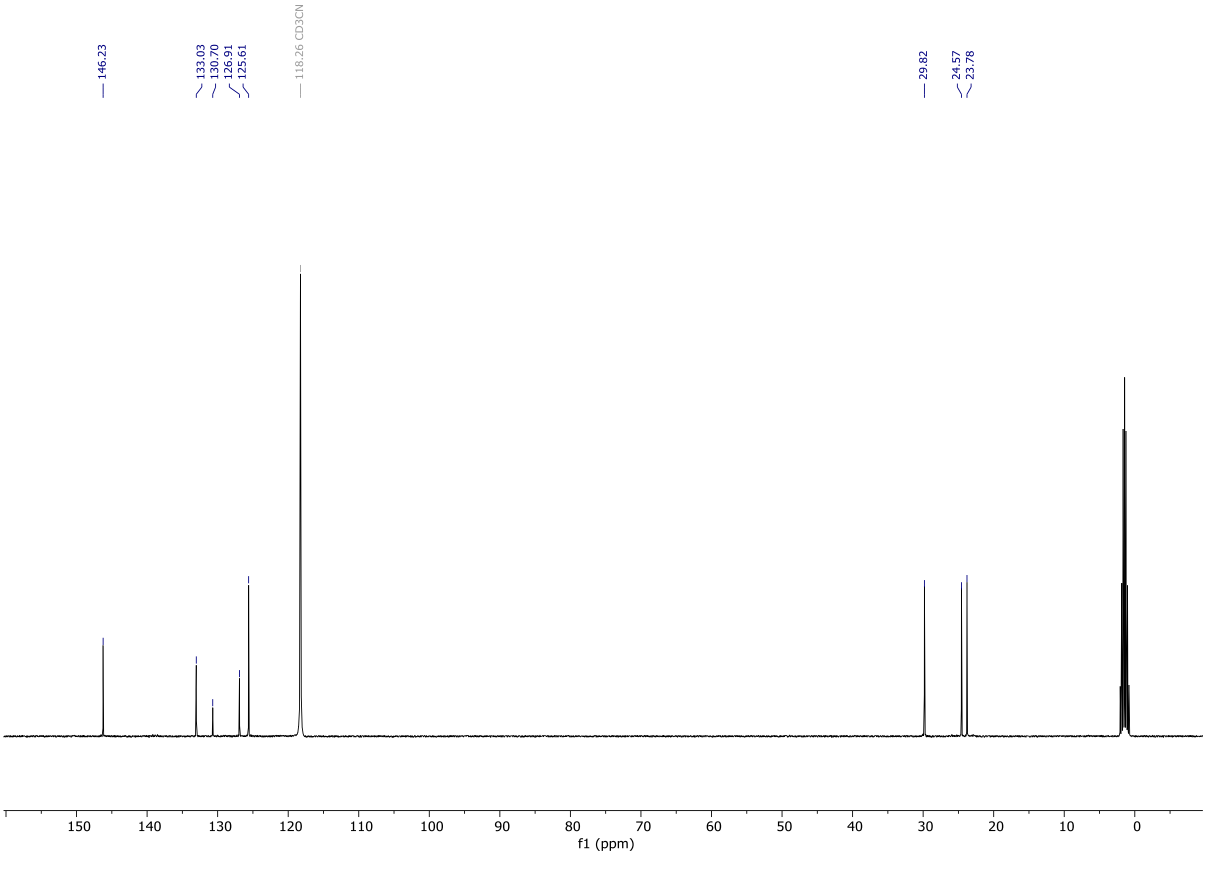

Figure S 39: ^{13}C NMR of compound **IDippAlI$_4$.**

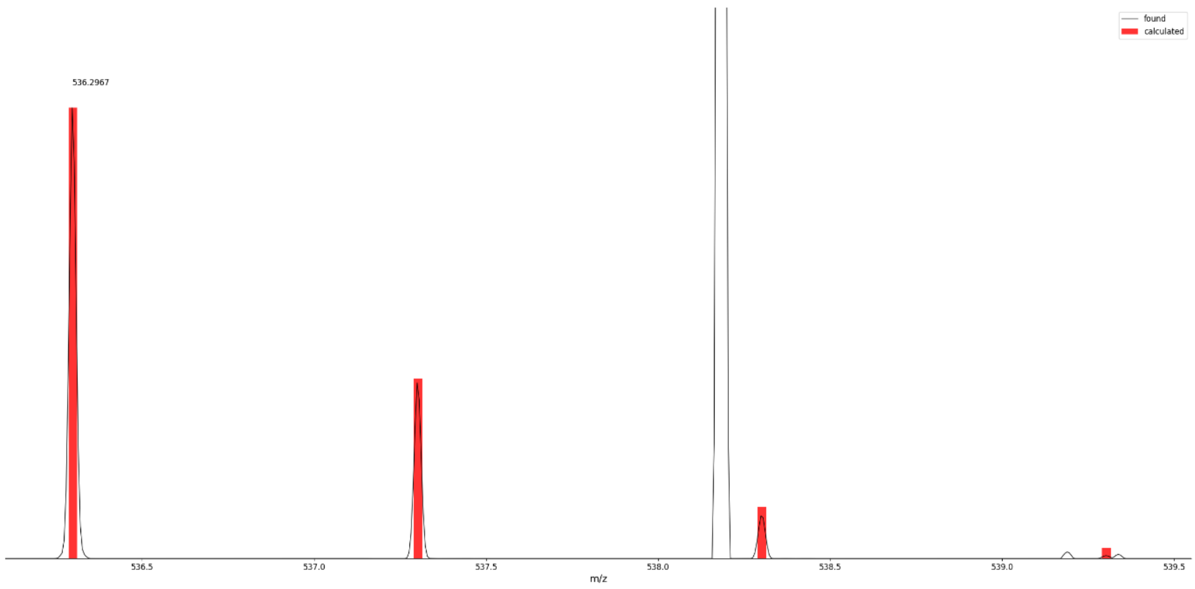

Figure S 40: LIFDI-MS spectrum for compound **2**.

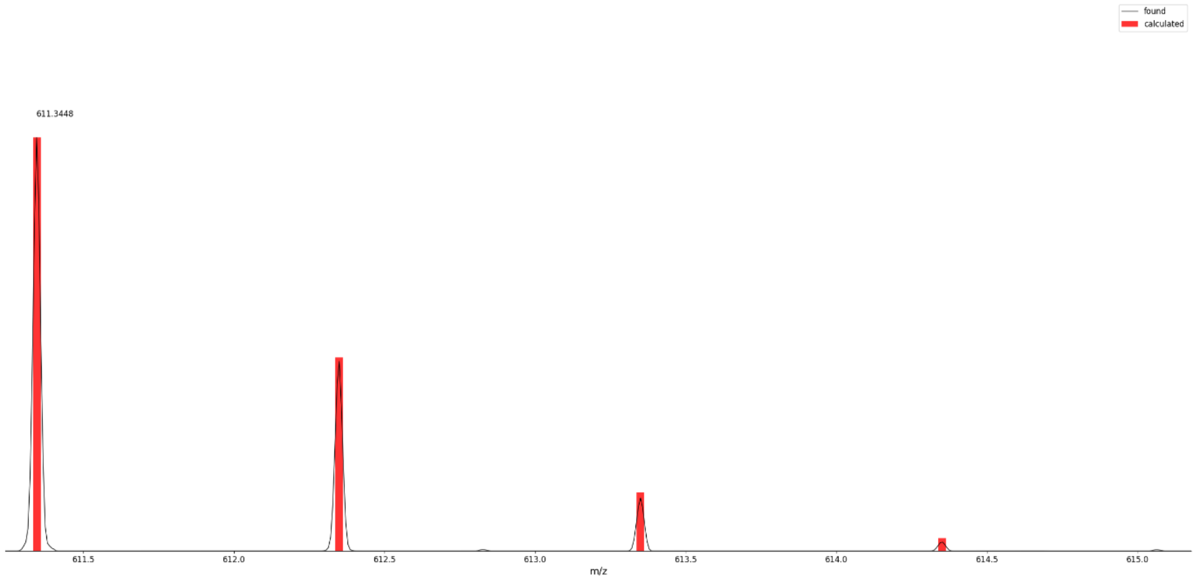

Figure S 41: LIFDI-MS spectrum for compound **3**.

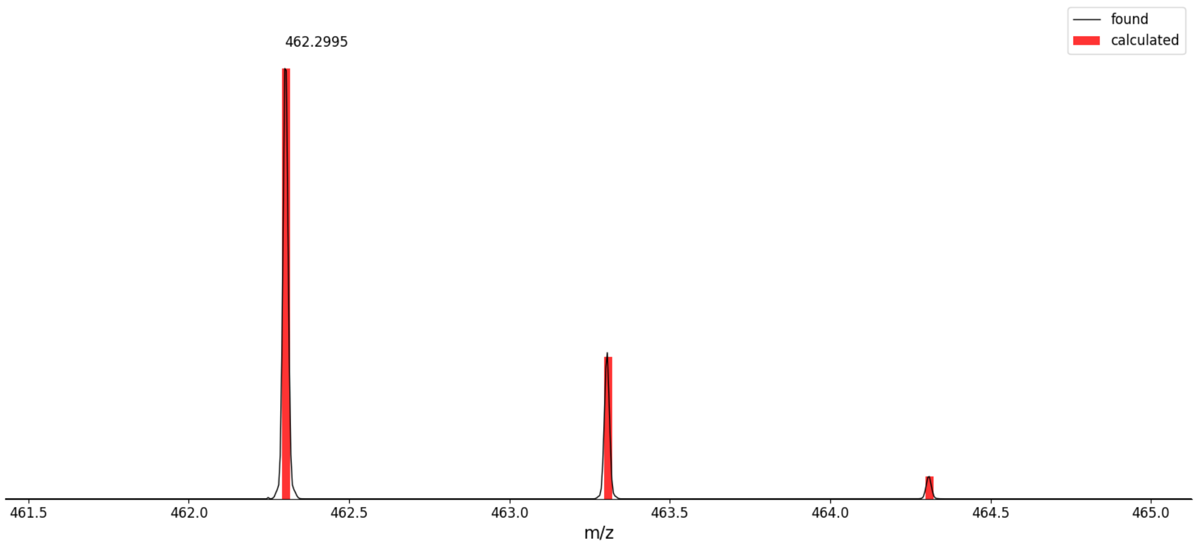

Figure S 42*:* LIFDI-MS spectrum for compound **4a** (+2H, - $AlCl_3$ - $SiMe_3$).

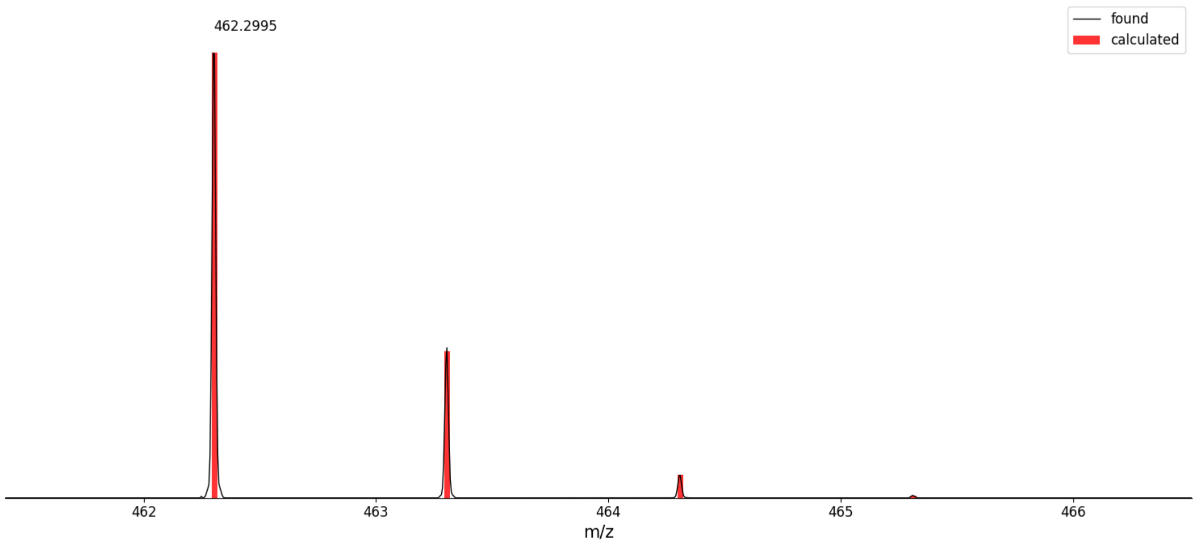

Figure S 43*:* LIFDI-MS spectrum for compound **4b** (+2H, - $AlCl_3$ - $SiMe_3$).

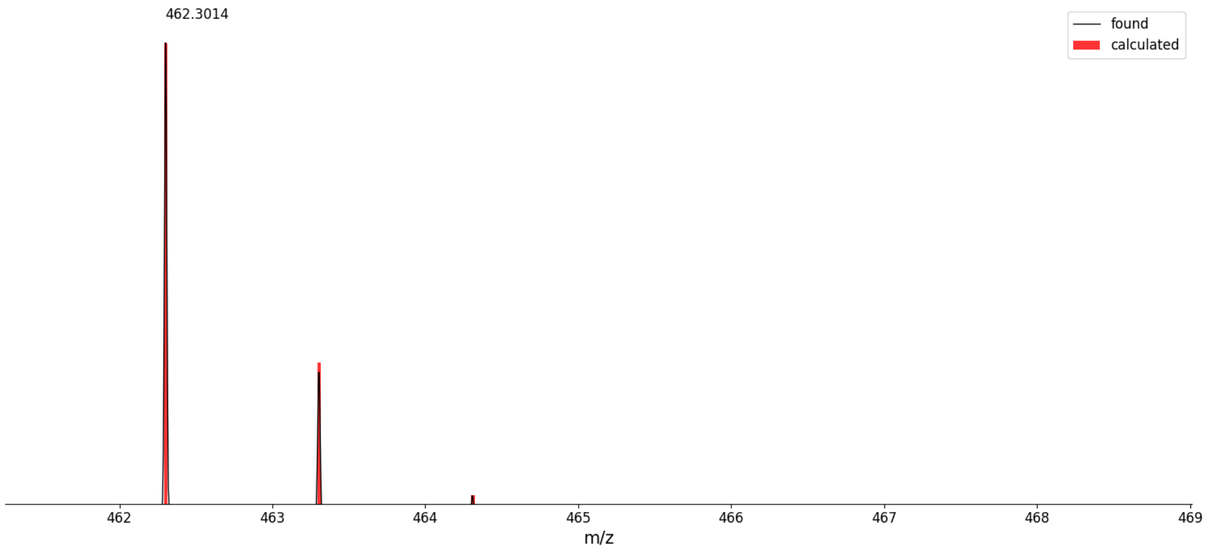

Figure S 44: LIFDI-MS spectrum for compound **5a** (+2H, - $AlCl_3$ - $SiMe_3$).

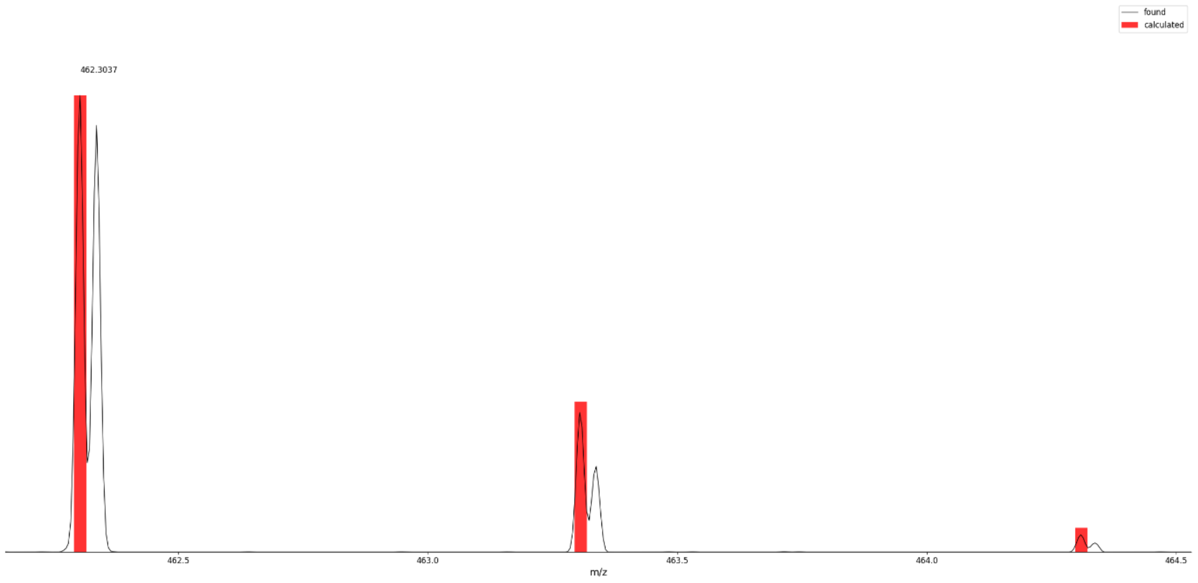

Figure S 45: LIFDI-MS spectrum for compound **5b** (+2H, - $AlCl_3$ - $SiMe_3$).

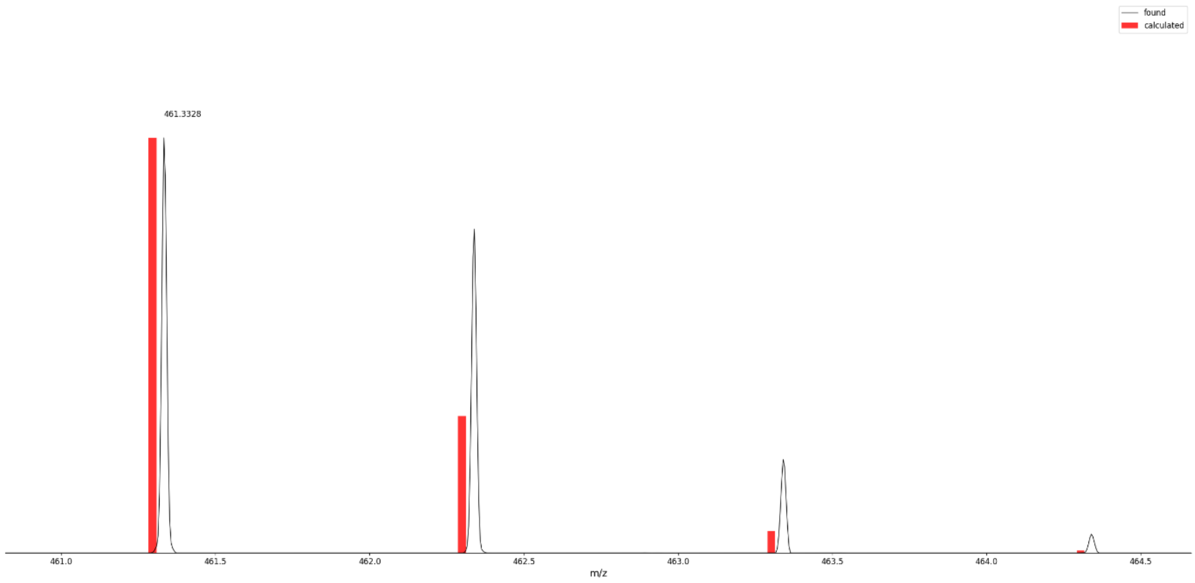

Figure S 46: LIFDI-MS spectrum for compound **5c** (+H, - $AlCl_3$ - $SiMe_3$).

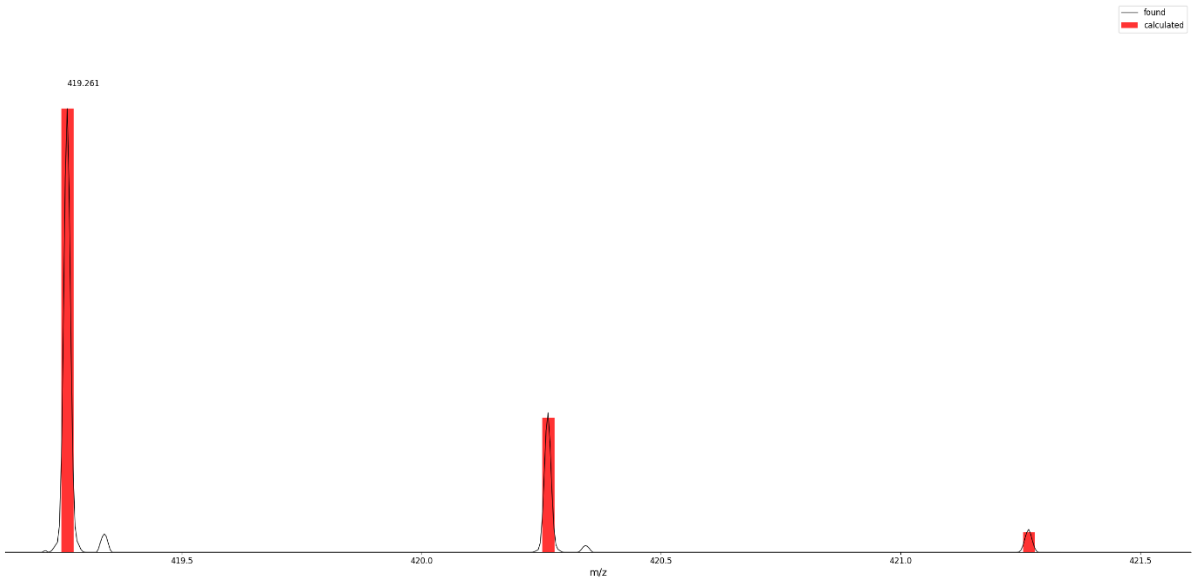

Figure S 47: LIFDI-MS spectrum for compound **6** (-BCl^{m}Ter).

Crystallographic Details

Table S1 Crystallographic details

	**2**	**3**	**4a**
CCDC number	**2505556**	**2505557**	**2505558**
Empirical formula	$C_{31}H_{45}N_2O_2PSi$	$C_{37}H_{50}N_3OPSi$	$C_{36}H_{54}AlCl_3N_5PSi$
Formula weight	536.75	611.86	749.23
Temperature [K]	100.00	100(2)	100.00
Crystal system	tetragonal	triclinic	monoclinic
Space group (number)	$P4_1$ (76)	$P\overline{1}$ (2)	$P2_1/n$ (14)
a [Å]	14.0694(8)	11.2293(4)	15.6478(8)
b [Å]	14.0694(8)	11.5685(5)	13.2259(7)
c [Å]	16.0170(10)	15.9164(6)	20.4075(12)
α [°]	90	111.0810(10)	90
β [°]	90	96.9690(10)	98.748(2)
γ [°]	90	104.9070(10)	90
Volume [Å^3]	3170.5(4)	1811.13(12)	4174.3(4)
Z	4	2	4
ρ_{calc} [gcm^{-3}]	1.124	1.122	1.192
μ [mm^{-1}]	0.153	0.140	0.338
F(000)	1160	660	1592
Crystal size [mm^3]	0.143×0.15×0.365	0.281×0.431×0.588	0.212×0.400×0.938
Crystal colour	clear light yellow	Light yellow	colourless
Crystal shape	block	block	block
Radiation	MoK_α (λ=0.71073 Å)	MoK_α (λ=0.71073 Å)	MoK_α (λ=0.71073 Å)
2θ range [°]	5.79 to 51.31 (0.82 Å)	3.83 to 51.45 (0.82 Å)	4.04 to 51.42 (0.82 Å)
Index ranges	−17 ≤ h ≤ 17	−13 ≤ h ≤ 13	−19 ≤ h ≤ 19
	−17 ≤ k ≤ 17	−14 ≤ k ≤ 14	−16 ≤ k ≤ 16
	−19 ≤ l ≤ 19	−19 ≤ l ≤ 19	−24 ≤ l ≤ 24
Reflections collected	49885	35970	173347
Independent reflections	5949	6888	7775
	R_{int} = 0.0509	R_{int} = 0.0265	R_{int} = 0.0360
	R_{sigma} = 0.0281	R_{sigma} = 0.0180	R_{sigma} = 0.0138
Completeness to θ = 25.242°	99.8 %	100.0 %	98.5 %
Data / Restraints / Parameters	5949 / 1 / 345	6888 / 0 / 399	7775 / 0 / 438
Absorption correction T_{min}/T_{max} (method)	0.6999 / 0.7453 (none)	0.7178 / 0.7453 (multi-scan)	0.7108 / 0.7453 (multi-scan)
Goodness-of-fit on F^2	1.046	1.100	1.091
Final *R* indexes [I≥2σ(I)]	R_1 = 0.0289<br>wR_2 = 0.0740	R_1 = 0.0335<br>wR_2 = 0.0754	R_1 = 0.0383<br>wR_2 = 0.0927
Final *R* indexes [all data]	R_1 = 0.0319<br>wR_2 = 0.0751	R_1 = 0.0397<br>wR_2 = 0.0813	R_1 = 0.0433<br>wR_2 = 0.0978
Largest peak/hole [eÅ^{-3}]	0.41/−0.17	0.26/−0.27	0.71/−0.40
Flack X parameter	0.01(2)		

Table S2 Crystallographic details

	**4b**	**5c**	**6**
CCDC number	**2505559**	**2505561**	**2505562**
Empirical formula	$C_{36}H_{54}AlBr_3N_5PSi$	$C_{39}H_{67}IN_3OPSi_2$	$C_{57}H_{74}BClN_3OP$
Formula weight	882.61	808.00	894.42
Temperature [K]	100.00	100.00	100(2)
Crystal system	tetragonal	monoclinic	monoclinic
Space group (number)	$P4_32_12$ (96)	$C2/m$ (12)	$P2_1/n$ (14)
a [Å]	12.6303(11)	15.8020(17)	13.2836(11)
b [Å]	12.6303(11)	17.4059(17)	15.9894(13)
c [Å]	52.324(6)	16.5125(16)	25.1257(18)
α [°]	90	90	90
β [°]	90	103.159(4)	96.444(3)
γ [°]	90	90	90
Volume [$Å^3$]	8346.9(18)	4422.5(8)	5302.9(7)
Z	8	4	4
ρ_{calc} [gcm^{-3}]	1.405	1.214	1.120
μ [mm^{-1}]	3.019	0.845	0.142
F(000)	3616	1704	1928
Crystal size [mm^3]	0.273×0.289×0.409	0.088×0.120×0.212	0.169×0.198×0.286
Crystal colour	yellow	colourless	yellow
Crystal shape	block	block	plate
Radiation	MoK_α (λ=0.71073 Å)	MoK_α (λ=0.71073 Å)	MoK_α (λ=0.71073 Å)
2θ range [°]	3.98 to 51.49 (0.82 Å)	4.68 to 51.36 (0.82 Å)	4.19 to 51.42 (0.82 Å)
Index ranges	−15 ≤ h ≤ 15<br>−15 ≤ k ≤ 15<br>−63 ≤ l ≤ 63	−19 ≤ h ≤ 19<br>−21 ≤ k ≤ 21<br>−20 ≤ l ≤ 20	−16 ≤ h ≤ 16<br>−19 ≤ k ≤ 19<br>−30 ≤ l ≤ 30
Reflections collected	250574	32379	87380
Independent reflections	7979<br>R_{int} = 0.0531<br>R_{sigma} = 0.0176	4341<br>R_{int} = 0.0558<br>R_{sigma} = 0.0358	10081<br>R_{int} = 0.1102<br>R_{sigma} = 0.0640
Completeness to θ = 25.242°	99.9 %	99.6 %	99.9 %
Data / Restraints / Parameters	7979 / 0 / 383	4341 / 141 / 294	10081 / 0 / 610
Absorption correction T_{min}/T_{max} (method)	0.5426 / 0.7453<br>(multi-scan)	0.6946 / 0.7453<br>(none)	0.6789 / 0.7453<br>(multi-scan)
Goodness-of-fit on F^2	1.073	1.033	1.013
Final *R* indexes [$I \geq 2\sigma(I)$]	R_1 = 0.0295<br>wR_2 = 0.0755	R_1 = 0.0393<br>wR_2 = 0.0962	R_1 = 0.0485<br>wR_2 = 0.1038
Final *R* indexes [all data]	R_1 = 0.0317<br>wR_2 = 0.0766	R_1 = 0.0423<br>wR_2 = 0.0986	R_1 = 0.0843<br>wR_2 = 0.1208
Largest peak/hole [$eÅ^{-3}$]	0.40/−0.34	0.75/−0.68	0.28/−0.31
Flack X parameter	0.035(9)		

	**$IDippAlI_4$**
CCDC number	**2505560**
Empirical formula	$C_{31}H_{47}AlI_4N_2O$
Formula weight	998.28
Temperature [K]	100.00
Crystal system	monoclinic
Space group (number)	$C2/c$ (15)
a [Å]	28.806(2)
b [Å]	9.6799(7)
c [Å]	27.7171(18)
α [°]	90
β [°]	91.351(2)
γ [°]	90
Volume [Å^3]	7726.4(9)
Z	8
ρ_{calc} [gcm^{-3}]	1.716
μ [mm^{-1}]	3.273
F(000)	3840
Crystal size [mm^3]	0.169×0.216×0.304
Crystal colour	clear colourless
Crystal shape	plate
Radiation	MoK_α (λ=0.71073 Å)
2θ range [°]	4.44 to 51.41 (0.82 Å)
Index ranges	−35 ≤ h ≤ 34<br>−11 ≤ k ≤ 11<br>−33 ≤ l ≤ 33
Reflections collected	85119
Independent reflections	7327<br>R_{int} = 0.0364<br>R_{sigma} = 0.0186
Completeness to θ = 25.242°	99.7 %
Data / Restraints / Parameters	7327 / 0 / 363
Absorption correction T_{min}/T_{max} (method)	0.5903 / 0.7453 (analytical)
Goodness-of-fit on F^2	1.086
Final *R* indexes [I≥2σ(I)]	R_1 = 0.0202<br>wR_2 = 0.0475
Final *R* indexes [all data]	R_1 = 0.0214<br>wR_2 = 0.0480
Largest peak/hole [eÅ^{-3}]	0.96/−0.80
Extinction coefficient	0.000046(7)

References

S1 A. Doddi, D. Bockfeld, T. Bannenberg, P. G. Jones, M. Tamm, *Angew. Chem. Int. Ed.* **2014**, *53*, 13568-13572.

S2 M. Muhr, P. Heiß, M. Schütz, R. Bühler, C. Gemel, M. H. Linden, H. B. Linden, R. A. Fischer, *Dalton Trans.* **2021**, *50*, 9031-9036.

S3 Bruker, 2015.5-2 ed., Bruker AXS Inc, Madison, Wisconsin, USA, **2015**.

S4 Bruker, Version 8.40A ed., Bruker AXS Inc., Madison, Wisconsin, USA, **2016**.

S5 Bruker, Version 2016/2, ed., Bruker AXS Inc., Madison, Wisconsin, USA, **2016**.

S6 G. M. Sheldrick, *Acta Crystallographica Section C: Crystal Structure Communications* **2015**, *71*, 3-8.

S7 G. M. Sheldrick, *Acta Crystallographica Section A: Foundations of Crystallography* **2015**, *71*, 3-8.

S8 C. B. Huebschle, G. M. Sheldrick, B. Dittrich, *J. Appl. Cryst.* **2011**, *44*, 1281.

S9 A. Spek, *Acta Crystallographica Section C* **2015**, *71*, 9-18.

S10 G. M. Sheldrick, University of Göttingen, Göttingen, Germany, **2014**.

S11 A. J. C. Wilson, V. Geist*, Vol. C*, Kluwer Academic Publishers (published for the International Union of Crystallography), Dordrecht/Boston/London, **1992**, pp. Tables 6.1.1.4 (pp 500-502), 504.502.506.508 (pp. 219-222) and 504.502.504.502 (pp. 193-199).

S12 A. L. Spek, Utrecht University, Utrecht, Netherlands **2010**.

S13 C. F. Macrae, I. J. Bruno, J. A. Chisholm, P. R. Edgington, P. McCabe, E. Pidcock, L. Rodriguez-Monge, R. Taylor, J. van de Streek, P. A. Wood, *J. Appl. Crystallogr.* **2008**, *41*, 466-470.

S14 D. Kratzert. *FinalCif*; D. Kratzert. https://dkratzert.de/finalcif.html.
